# A Scalable Multi-Sensor Vision Framework for Automated Bat Monitoring and 3D Habitat Analysis

**DOI:** 10.3390/s26175446

**Published:** 2026-08-28

**Authors:** José-Angel Arroyo-Romero, Isabel Bárcenas-Reyes, Juan-Bautista Hurtado-Ramos, Francisco-Javier Ornelas-Rodríguez, Erick-Alejandro González-Barbosa, Alfonso Ramirez-Pedraza, José-Joel González-Barbosa

**Affiliations:** 1 CICATA-Unidad Queretaro, Instituto Politécnico Nacional, Queretaro 76090, Mexico; jarroyor2100@alumno.ipn.mx (J.-A.A.-R.); jbautistah@ipn.mx (J.-B.H.-R.); fornelasr@ipn.mx (F.-J.O.-R.); aramirez_ixm@ipn.mx (A.R.-P.); 2 Facultad de Ciencias Naturales, Universidad Autónoma de Queretaro, Queretaro 76230, Mexico; isabel.barcenas@uaq.mx; 3 Tecnológico Nacional de México/ITS de Irapuato, Guanajuato 36821, Mexico; erick.gb@irapuato.tecnm.mx

**Keywords:** bats, near infrared images, RGB images, YOLO, vision system

## Abstract

Automated wildlife monitoring systems are essential for studying bat populations in natural environments, where nocturnal behavior, high flight speeds, and limited illumination make conventional observation difficult. This paper presents a modular multi-sensor vision system that integrates RGB, near-infrared (NIR), and depth cameras for automated bat monitoring. The proposed architecture consists of one main module and two secondary modules that can be configured into multiple operating modes according to monitoring requirements. The main module operates independently to perform real-time habitat reconstruction using an integrated depth camera or bat detection using a YOLO-based model. When combined with one secondary module, it forms a stereo vision system for three-dimensional localization; when combined with both secondary modules, it generates panoramic images that substantially expand the field of view for monitoring large cave entrances and other complex environments. The proposed modular architecture enables flexible deployment while supporting multiple sensing configurations within a single platform. The modular design provides scalability, geometric consistency through multi-sensor calibration, and flexible deployment, enabling accurate bat detection, habitat reconstruction, and wide-area monitoring within a unified sensing framework. The proposed system provides a versatile and scalable solution for adapting wildlife monitoring to different environmental conditions and observation scenarios. Experimental results demonstrate a detection precision of 0.893, a panoramic field of view of 119°, and real-time processing at 60 fps, validating the effectiveness of the proposed modular architecture.

## 1. Introduction

Several studies on landscape ecology associated with bats have linked the geographical distribution of these mammals to the availability of natural or artificial roosts located near their primary food sources [1,2,3]. A clear example is the common vampire bat, *Desmodus rotundus*, which feeds exclusively on blood, primarily that of cattle [3,4]. This species is also recognized as the principal natural reservoir of the rabies virus in the Americas. Due to its hematophagous feeding behavior, it facilitates the transmission of the pathogen to livestock, causing bovine paralytic rabies (BPR), a zoonotic disease that also represents a significant public health concern. Another example is the insectivorous bat *Tadarida brasiliensis*. This species provides important ecological benefits by consuming large quantities of insects considered agricultural pests, thereby contributing to a reduction in the use of agrochemicals in grazing areas [5]. However, *Diphylla ecaudata* has been reported to share roosts with the common vampire bat, *Desmodus rotundus*, illustrating the complex ecological interactions that can occur among bat species within shared habitats [6,7]. Monitoring *Tadarida brasiliensis* is considered important, as it is also a reservoir of the rabies virus due to its coexistence with *Desmodus rotundus* individuals belonging to colonies infected with the virus [4,8].

In Mexico, surveillance and control strategies for bovine paralytic rabies (BPR), implemented through the “National Campaign for the Prevention and Control of Rabies in Cattle and Other Livestock Species” (NOM-067-ZOO-2007), include the monitoring and population control of *Desmodus rotundus*. These efforts have revealed that the occurrence of BPR cases closely matches the geographical distribution of this bat species. As early as the 1960s, it was recognized that the distribution of *Desmodus rotundus* extended along the Gulf of Mexico coast from southern Tamaulipas to the Yucatán Peninsula and along the Pacific coast from southern Sonora to Chiapas. However, at that time, BPR was restricted to the Pacific coast and the states of Campeche and Yucatán. During the 1990s and 2000s, BPR spread to regions of the country where *Desmodus rotundus* was present but where the disease had not previously been reported. Currently, 26 of Mexico’s 32 federal states are considered endemic for both BPR and *Desmodus rotundus* [4,9]. Furthermore, it has been observed that the geographical distribution of *Desmodus rotundus* is not constrained by climatic conditions or altitude, but rather by the topography of certain regions of Mexico, which provides suitable roosting sites for the survival of the species and the establishment of its colonies. These colonies are increasingly found in close proximity to human settlements and livestock production areas [4].

Data on the presence of hematophagous bats, *Desmodus rotundus*, and insectivorous bats, particularly *Tadarida brasiliensis*, are generally obtained through epidemiological surveillance activities aimed at controlling vampire bats in natural roosts and livestock enclosures following the notification of rabies outbreaks in cattle. In addition, some bats are submitted for diagnostic laboratory testing for rabies. Consequently, these monitoring strategies introduce a statistical bias, as they do not account for healthy bats that have already left the roost, resulting in an incomplete representation of the population. Visual bat-counting techniques have proven useful for monitoring bat populations; however, they tend to underestimate population data because they do not account for the ecological behavior of bats throughout different seasons of the year [10]. For example, some bat species preferentially occupy deep rock crevices during hibernation or the summer breeding season, whereas other species use these shelters for mating and migration [11,12]. This behavioral variability, combined with the inherent limitations of conventional epidemiological surveillance, underscores the need for monitoring approaches capable of capturing population dynamics beyond isolated observations tied to disease-outbreak events.

In recent years, the use of vision-based monitoring systems has increased considerably due to advances in computational technologies and the development of sensors with enhanced capabilities. These systems have been widely adopted in various fields, including robotics [13,14,15,16,17] and security systems [18,19,20,21]. In the field of biological sciences, computer vision technologies have also been employed for tracking and monitoring a wide variety of species, both in urban environments [22,23] and in their natural habitats [24,25,26]. In different regions around the world, bat-monitoring systems based on optical, acoustic, and thermal sensors have been developed, enabling the automation of detection and analysis tasks for these mammals. These technological developments have been designed under the premise of minimal human intervention in low-light conditions or complex subterranean environments, such as caves, hollow trees, abandoned buildings, sewers, bridges, and rock crevices, among others. Such systems have been employed for monitoring bats with the objectives of counting, classification, species identification, and flight behavior analysis [27,28,29,30].

In [31], a stereo camera system was used to record bat emergence from cave entrances, combining motion detection and 3D reconstruction to analyze flight trajectories and estimate emergence rates without disturbing the animals. Similarly, refs. [32,33] employed thermal images acquired using UAV (Unmanned Aerial Vehicle)-based platforms to monitor bats over large and inaccessible areas, where deep learning was used to estimate population counts from thermal signatures under challenging conditions. In [34], a camera trap combining infrared video, RGB imaging, and white flash is proposed for bat species identification using deep neural networks (BatNet), achieving classification of up to 13 species under controlled conditions with enhanced contrast for segmentation. It achieved an overall accuracy of 99.3% on images from trained sites, with precision, recall, and F1-score values ranging from 0.97 to 1.00 across the 13 evaluated species. At untrained sites, the accuracy remained high (96.7–98.2%), but it was lower at sites with atypical viewing angles or distances (as low as 17.8%). For ecological studies, the model detected all species identified by expert observers.

Regarding deployment settings, monitoring systems are typically implemented either inside caves or in open environments. For instance, Ref. [35] exploited the stable environmental conditions inside caves to improve segmentation-based bat counting. In contrast, ref. [36] addressed the challenges of open-air monitoring by combining background modeling, temporal association of detections, and deep learning-based counting, achieving reliable estimates for scenes containing up to 11 bats per image. Similarly, ref. [37] employed a multi-camera system positioned at cave entrances to monitor emerging bats, using a U-Net-based model on grayscale and blue-channel images to improve contrast and segmentation robustness under varying illumination conditions. In [38] thermal imaging was combined with ultrasonic recordings for multimodal bat identification, whereas ref. [39] extended bat monitoring to agricultural environments, supporting ecological management through detection, counting, and tracking for biodiversity conservation.

Characterizing bat-inhabited caves using artificial intelligence techniques based on neural network algorithms can generate predictive models of potential roosts and estimate bat population abundance throughout the year. Such models can support the design and improvement of epidemiological surveillance strategies [40,41], including the scheduling of livestock vaccination campaigns and the monitoring of bat health through selective colony management and the identification of potential migration routes under the One Health framework [42]. Therefore, the implementation of artificial intelligence algorithms can contribute to improved monitoring of bat populations and their roosts, enabling a more accurate estimation of the risk of rabies transmission to cattle, humans, and other animal species. The systematic collection of monitoring data facilitates the implementation of sanitary measures such as targeted vaccination, selective colony management, and the issuance of early warnings in high-risk areas. Furthermore, it contributes to a better understanding of the relationship between environmental factors and vector behavior, promoting a comprehensive approach that balances public health protection, a reduction in economic losses in the livestock sector, and ecosystem conservation.

This work presents the development of a flexible multi-sensor computer vision system for monitoring and analyzing bat behavior in their natural habitats. Because bat roosts exhibit diverse geometries and are often difficult to access, the proposed system adopts a modular, portable, and easy-to-deploy architecture that can be adapted to different cave sizes, morphologies, and monitoring requirements. The platform supports four complementary operating modes: (i) dynamic 3D reconstruction of cave environments for habitat mapping and survey planning; (ii) the Near-Infrared Stereo Imaging Platform (NISIP) for estimating the three-dimensional position, size, morphology, and flight trajectories of bats; (iii) real-time bat detection using a deep learning model optimized for low-light conditions; and (iv) the Wide-Field Near-Infrared Imaging Platform, which combines multiple camera views to increase the field of view, improve continuous tracking, and enable the simultaneous monitoring of multiple bats with complex flight patterns trajectories.

## 2. Methodology

**Figure 1:**
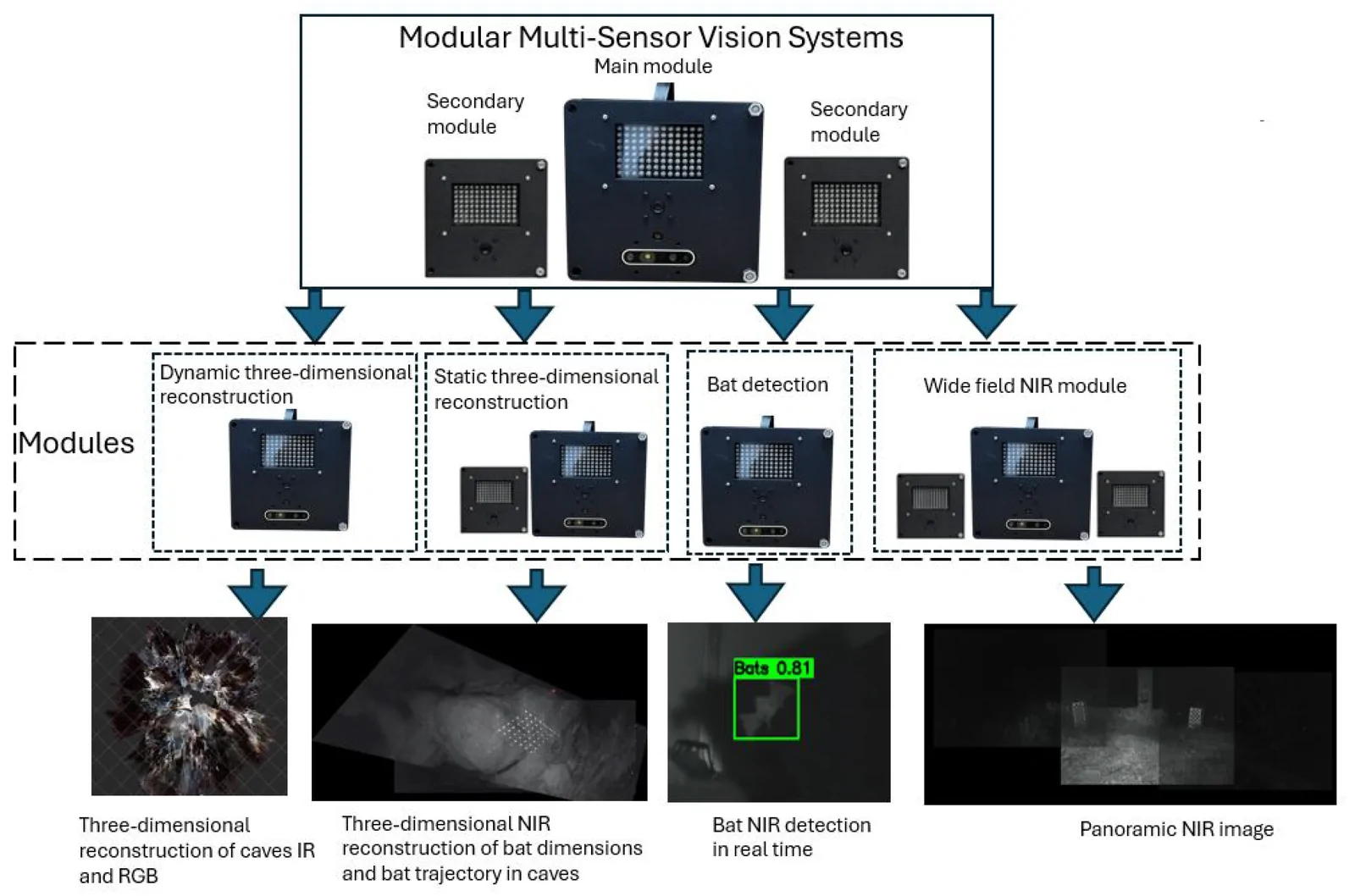
The figure illustrates the possible module configurations and the corresponding output image types. The main module operates either independently for 3D scene reconstruction or bat detection, or in combination with one or two secondary modules to enable stereo imaging or expanded field-of-view acquisition for large-area habitat monitoring.

A modular system composed of a main module and two secondary modules, as shown in Figure 1, is presented for the study, detection, and 3D analysis of bats and their habitats in cave environments. Cave entrances where bats roost show high geometric variability: in some cases, narrow entrances allow a single camera to adequately cover bat flow, while in other cases, wider entrances require multiple sensors to fully capture entry and exit trajectories. For this reason, the system is designed with a modular architecture that enables the field of view to be extended by adding additional modules, adapting to the specific characteristics of each cave. The platform also supports 3D reconstruction of the cave environment, which is useful for characterizing habitat structure, estimating bat movement paths, analyzing behavior patterns, and supporting ecological monitoring and conservation studies. The modular architecture, shown in Figure 1, is one of the main advantages of the system, providing flexibility and scalability. The main module can operate independently, performing specific tasks, or it can be integrated with the secondary modules to extend perception, reconstruction, and analysis capabilities of the full environment. This modularity allows the system to be optimally configured according to physical constraints of the environment, such as cave geometry.

**Figure 2:**
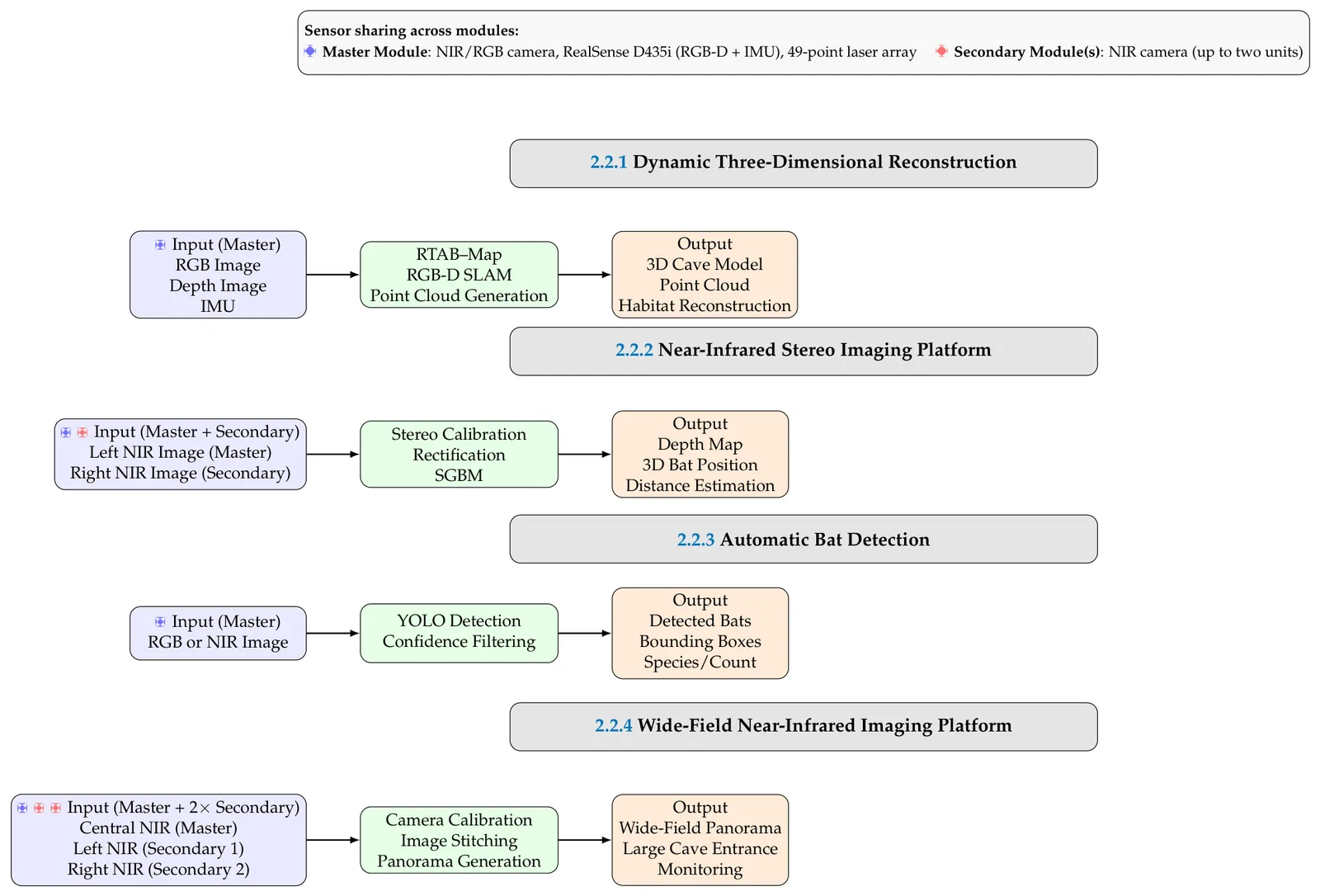
Workflow of the four operating modalities of the proposed modular multi-sensor monitoring platform. Color tags on each input block indicate which physical module(s) (master or secondary) supply the sensor data for that modality.

In this context, the system is designed to support four main operating modes, which are described below. The workflow shown in Figure 2 provides a visual overview of the mathematical models and processing pipeline employed in each operating mode.

1. Dynamic 3D environment reconstruction. Focused on generating 3D maps while the user moves through caves, using the model described in [43]. This mode supports habitat analysis, route planning, and field decision-making for bat observations (e.g., equipment and mist nets).
2. Near-Infrared Stereo Imaging Platform (NISIP). Uses a stereo setup from the main and two secondary modules with one camera to estimate depth and object dimensions. It provides accurate 3D positions, enabling trajectory and flight speed estimation from sequential positions, and supports morphological analysis (e.g., wingspan and body size).
3. Automatic bat detection. Based on [43], a detection model runs on the main module for real-time bat detection in low-light caves. Compared to [43], the dataset has been expanded and the YOLO version updated.
4. Wide-Field Near-Infrared Imaging Platform. Combines multiple cameras to increase field of view, improving bat localization and reducing tracking loss when individuals leave the field of view. Panoramic images enable multi-target detection and more stable tracking in complex flight patterns.

Integrating the detection model with the other system modes enables joint use of visual, spatial, and 3D information, improving overall behavioral analysis. Each module includes an infrared (IR) illumination system to light the scene without disturbing bats [44]. Modules also include local storage and internal batteries, ensuring autonomous and continuous operation in any configuration. This energy independence is essential for cave environments, where external power is limited or unavailable. Overall, the proposed modular architecture provides a robust, flexible, and scalable system for 3D monitoring, automatic detection, and behavioral analysis of bats in complex natural environments.

### 2.1. Module Description

The modular architecture allows the system to operate under different configurations, adapting to various acquisition and analysis scenarios. This section describes in detail the system’s operating configurations. The complete system has an approximate total weight of 6.5 kg, consisting of a 3.5 kg main module with dimensions of 22 × 22 × 14 cm and two secondary modules of 1.5 kg each, with dimensions of 18 × 18 × 11 cm.

All acquired data are stored locally on the embedded computer’s solid-state drive (SSD), since the target monitoring environments, such as caves and remote habitats, generally do not provide reliable wireless communication. The embedded computer performs real-time processing to enable immediate bat detection, habitat reconstruction, and system verification during field deployment. Compared with offline processing of raw sensor data, on-board processing allows operators to validate sensor calibration and acquisition quality in real time, reduces post-processing time, and supports future autonomous monitoring capabilities, while maintaining all raw data available for subsequent detailed analysis if required.

#### 2.1.1. Main Module

The main module has been designed to operate fully autonomously, integrating multiple functions oriented toward monitoring [43] and 3D analysis [26] of bat environments. The module includes a computer with 16 GB of RAM and a 512 GB hard drive running Ubuntu 20.04. Reconstruction is continuously generated while moving inside caves, enabling real-time 3D mapping using an Intel RealSense D435i camera (Intel Corporation, USA; manufactured in China/Thailand). In addition, the main module incorporates a near-infrared Arducam camera and an infrared illumination lamp. This near-infrared imaging system is used for stereo configuration, panoramic image acquisition, and automatic bat detection. To ensure portability and ergonomic handling during mapping tasks, the main module includes a handle located at the top, allowing the operator to safely and stably carry the system during cave exploration. The main module also includes a 49-laser array used for reconstruction and calibration. At the rear of the main module, a touchscreen is installed, allowing the user to intuitively select and configure the different operating modes of the system. In addition, the module includes four physical buttons for powering on the main computer, turning on the monitor, activating the laser system, and controlling the infrared lamp, respectively.

**Figure 3:**
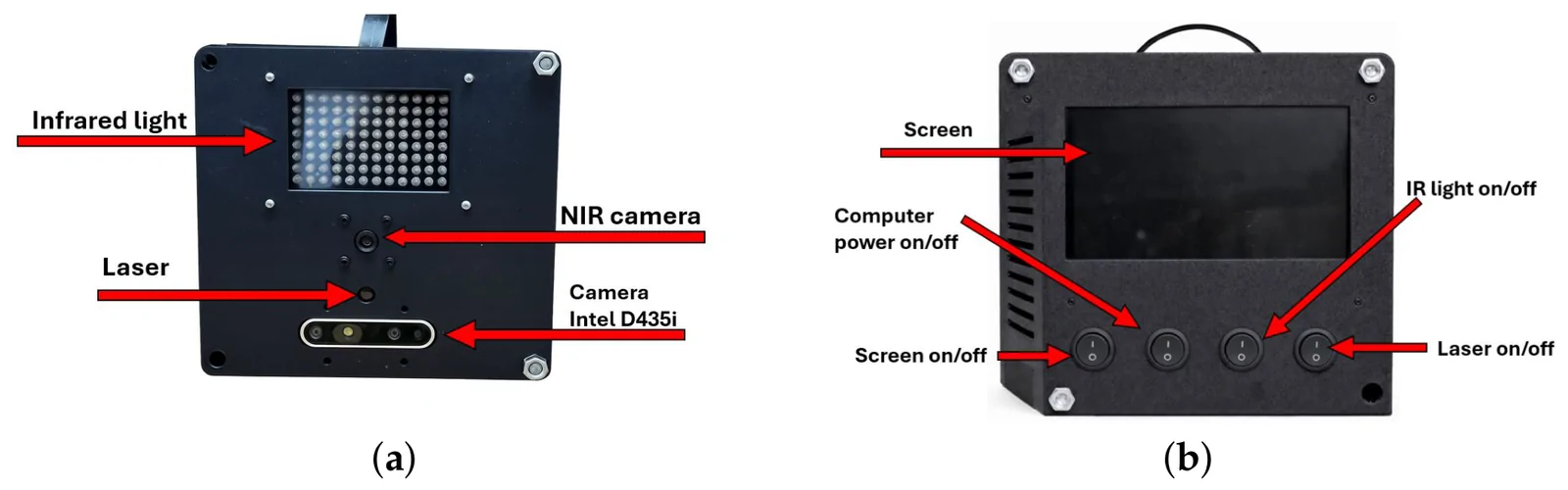
Front (**a**) and rear (**b**) views of the main module.

**Figure 4:**
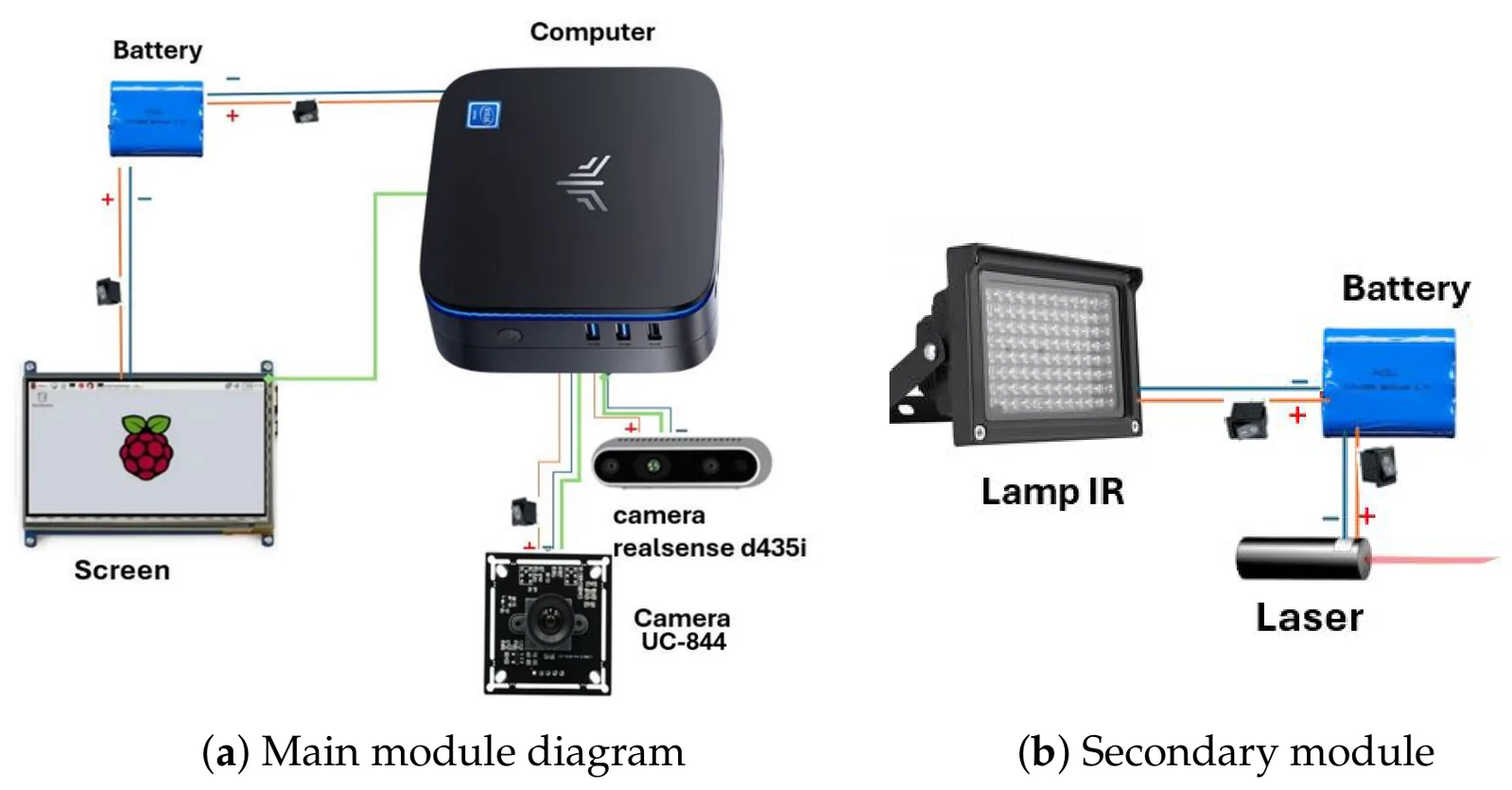
Diagrams of the main and secondary modules. (**a**) The main module comprises a computer with 16 GB RAM and a 512 GB SSD, a 7-inch LCD display (HDMI, 1024 × 600), an Intel RealSense D435i RGB-D camera with an IMU (1280 × 720 at 30 fps, 10 m depth range), and an Arducam UC844 camera (1280 × 720 at 60 fps). It is powered by a 19 V, 65 W battery. The illumination subsystem includes a 9 V infrared lamp and a 49-point laser array (6 V, 4.8 W). It is powered by a 9.6 V, 20 W Ni-MH battery.

Figure 3a shows the front view, which includes the infrared and depth cameras, as well as the IR lamp and laser array. Figure 3b shows the rear side, where the touchscreen and the power buttons for the different components can be observed. The electrical system is designed to ensure stable and autonomous operation of all electronic components. As shown in Figure 4a, the screen and the computing unit are powered by a 19 V 18 Ah rechargeable lithium-ion battery, which provides the necessary power for continuous operation of the main system. Both devices are directly connected to the computer, enabling power distribution and reducing losses associated with voltage conversion.

The energy efficiency of the system provides approximately 5.0 h of autonomy, thanks to the use of one pair of 19 V lithium rechargeable batteries and one pair of 9.6 V rechargeable batteries, in addition to a backup battery that extends field operation time, making it a compact, portable system suitable for use in hard-to-access environments such as natural caves. The infrared lamp and laser system are powered independently by a 9 V battery, which isolates their consumption from the main computing system. This configuration improves the electrical stability of the system and extends overall autonomy. The 9 V battery provides approximately 4 h of continuous operation for both the lamp and the laser, as shown in Figure 4b.

#### 2.1.2. Secondary Module

**Figure 5:**
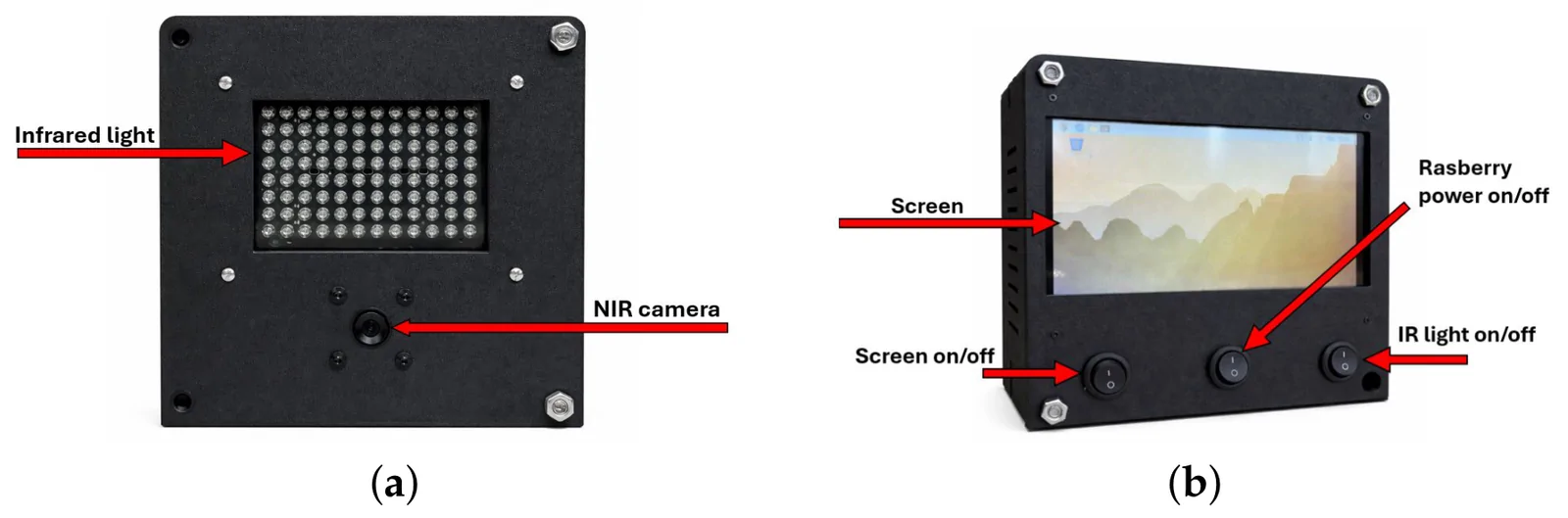
Front (**a**) and rear (**b**) views of secondary module.

The secondary module, as shown in Figure 5, is designed to complement the functions of the main module. It operates as a second camera (see Figure 5a) in a stereo configuration, increasing visual coverage and robustness against occlusions and lighting variations. This setup enables 3D reconstruction of the environment using synchronized image acquisition, which is processed to obtain complementary spatial information. Additionally, a laser-based reference system is used for in situ calibration of the stereo system. At the rear of the secondary module (see Figure 5b), a touchscreen is included, allowing the user to select the desired configuration for the module to operate as support for the main system. The module also includes three physical buttons for powering the Raspberry Pi, turning on the monitor, and controlling the infrared lamp switch, respectively. The module’s energy efficiency provides approximately 4.5 h of autonomy, which is important considering that bats typically leave their roosts around 9:30 p.m. and return around 11:57 p.m. [45]. This autonomy is achieved using a rechargeable 9 V lithium battery, making it a compact and lightweight device, suitable for integration with the main module and for use in hard-to-access environments.

**Figure 6:**
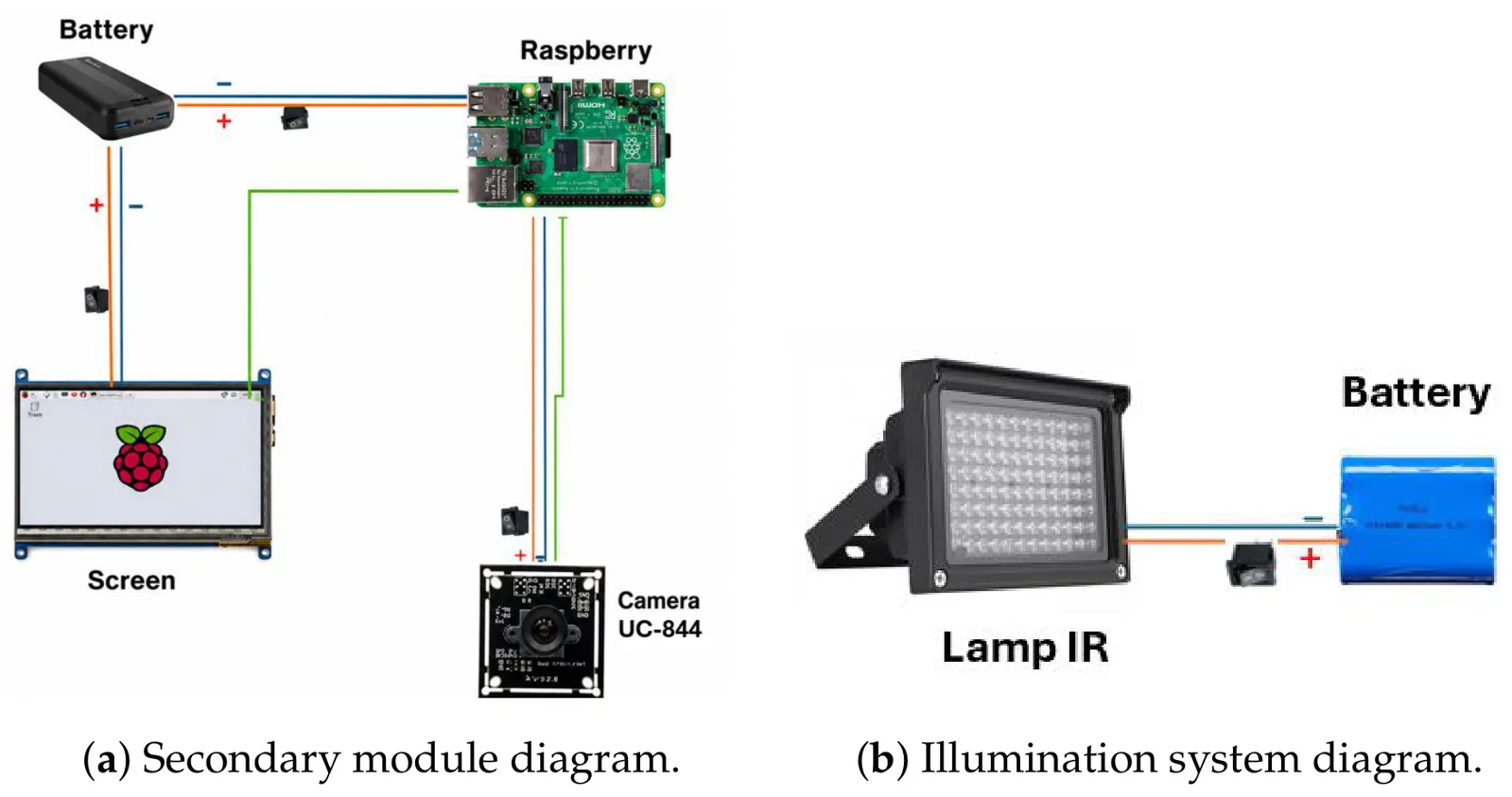
Panel (**a**) shows a diagram of the secondary module, consisting of a Raspberry Pi 5, a 7-inch HDMI LCD touchscreen with a resolution of 1024 × 600 pixels (5 V, 5 W), and an Arducam UC844 camera (1280 × 720 pixels at 60 FPS), all powered by a 5 V power bank. Panel (**b**) shows the illumination system, consisting of a 9 V IR lamp.

The electrical architecture, as shown in Figure 6, is based on the use of a 5 V power bank, which supplies power to both the display and the Raspberry Pi, serving as the main power source for the module. Following the approach adopted in different compact applications using the Raspberry Pi [46,47], the peripheral devices connected to the system, including the camera, display, and hard drive, are powered directly from the Raspberry Pi. This enables efficient power distribution and simplifies the internal wiring of the equipment (see Figure 6a). This configuration reduces the need for additional voltage regulators and contributes to improving the overall energy efficiency of the system. The infrared lamp is powered by a dedicated 9 V battery, allowing its power consumption to be isolated from the rest of the system (see Figure 6b). This battery provides up to 4 h of continuous operation for the near-infrared (NIR) camera, ensuring the stable performance of this component throughout the data acquisition sessions.

### 2.2. Mathematical Model of the System Operating Configurations

For the correct operation of the system, the integrated modules require precise calibration. Properly calibrated sensors ensure reliable information. This section describes the methodology used for the calibration and coordination of the main and secondary modules, both in individual operation and in joint operation, for each system operating configuration.

#### 2.2.1. Dynamic Three-Dimensional Reconstruction of the Environment

A dynamic reconstruction of the environment inhabited by the bats is performed using RTAB-Map (Real-Time Appearance-Based Mapping) within Robot Operating System (ROS), following the methodology presented in [48]. RTAB-Map is a visual Simultaneous Localization and Mapping (SLAM) algorithm designed for real-time three-dimensional map construction through appearance-based loop closure detection [49]. Its implementation in ROS enables the integration of sensors such as RGB-D cameras, LiDAR, and stereo vision systems for applications in mobile robotics and 3D reconstruction. The RTAB-Map approach is based on the extraction of visual features from images acquired by the sensor, which are used to generate visual signatures. These signatures are stored in a three-level memory architecture consisting of Short-Term Memory (STM), Working Memory (WM), and Long-Term Memory (LTM). This architecture limits the computational growth of the system while maintaining real-time performance [50]. The localization and mapping process relies on the incremental estimation of the robot’s trajectory using visual or sensor-based odometry. Subsequently, feature matching and geometric verification techniques are employed to detect loop closures, enabling the correction of accumulated errors through pose graph optimization. In ROS, RTAB-Map is implemented as a collection of nodes that manage data acquisition, odometry estimation, map construction, and visualization. This integration facilitates its use in applications such as autonomous navigation, environment inspection, and three-dimensional reconstruction of complex spaces.

#### 2.2.2. Near-Infrared Stereo Imaging Platform (NISIP)

A modular infrared stereo vision system was developed to obtain three-dimensional features of the environment, enabling the estimation of the bats’ velocities and dimensions. The stereo system consists of two independent cameras, providing greater versatility to the modular architecture of the system. To operate in stereo mode, the system can be configured in two ways. The first consists of fixing the modules at the working site and acquiring images using a calibration pattern to perform the geometric calibration of the stereo system. However, this option is not always feasible in field environments, as the use of calibration patterns may be impractical. An alternative would be to maintain the stereo system in a previously calibrated fixed configuration, although this reduces the flexibility of the modular system. Therefore, a different solution was adopted by incorporating an array of 49 lasers into the main module. When projected onto a surface within the working environment, these laser points can be used both for the three-dimensional reconstruction of the illuminated object or surface and for the calibration of the stereo system. In this way, once the modules have been positioned, it is only necessary to project the laser pattern onto a surface visible to both cameras and perform the calibration process. It should be noted that this surface may be either planar or irregular. The proposed model describes a two-camera infrared stereo system with variable relative distance and orientation, observing a set of 49 laser points projected from one of the cameras with known positions. Each laser point generates a stereo correspondence, which, together with the previously calibrated intrinsic and extrinsic parameters, allows its three-dimensional position to be recovered through triangulation.

Equation (1) represents the stereo triangulation process through the three-dimensional reconstruction of the laser points projected onto the environment. In particular, the function C(·) denotes the 3D reconstruction operator, which computes the position of the point $P_{W}^{\left(k\right)}$ in space from the projection matrices of both cameras and the image-plane correspondences $p_{L}^{\left(k\right)}↔p_{R}^{\left(k\right)}$. The reconstruction is based on the intersection of the projection rays generated from each camera toward the observed point, using the intrinsic parameters $K_{L}$, $K_{R}$ and the extrinsic relationship $\left(R_{RL},t_{RL}\right)$ between the two cameras.

(1) $$P_{W}^{\left(k\right)}=C\left(K_{L}\left[I|0\right],K_{R}\left[R_{RL}|t_{RL}\right],p_{L}^{\left(k\right)},p_{R}^{\left(k\right)}\right),\;k=1,\ldots ,49$$

(2) $$Z^{\left(k\right)}=\hat{C}\;\left(p_{L}^{\left(k\right)},p_{R}^{\left(k\right)},R_{RL},t_{RL}\right)$$

where $K_{L,R}$ denotes the intrinsic matrices, $\left(R_{RL},t_{RL}\right)$ represents the relative pose between the cameras, and $p_{L}^{\left(k\right)}↔p_{R}^{\left(k\right)}$ denotes the correspondences of the 49 laser points. Equation (2), which represents the depth triangulation process, computes the depth information from the corresponding points in the left and right images, $\left(p_{L}^{\left(k\right)},p_{R}^{\left(k\right)}\right)$, together with their relative extrinsic parameters, $R_{RL}$ and $t_{RL}$.

##### NISIP Calibration

**Figure 7:**
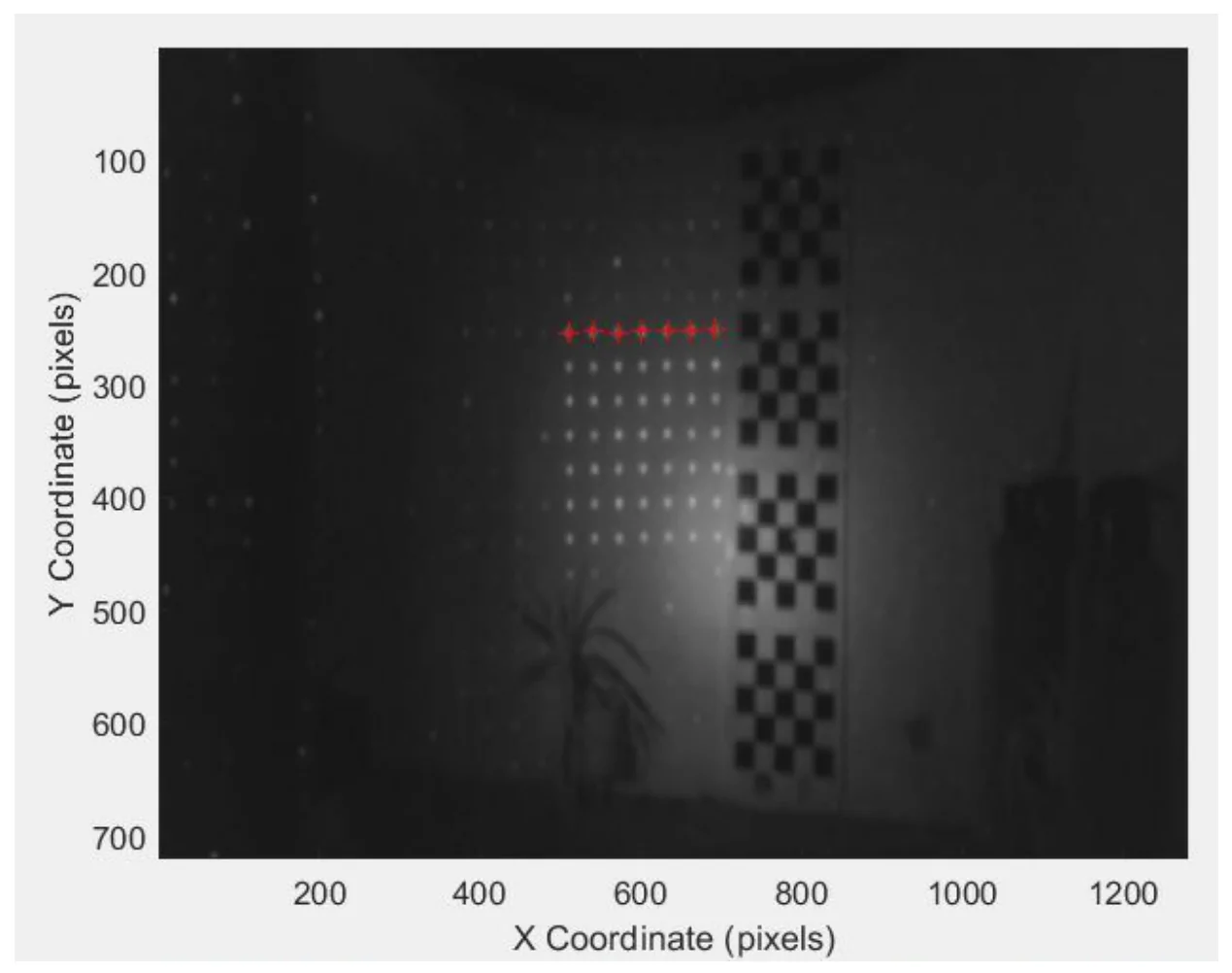
Typical calibration image of the 49-point laser array projected onto a checkerboard calibration target. Four images are sufficient to estimate the laser-to-camera calibration.

The 49-laser array is calibrated with respect to the main camera under controlled laboratory conditions, since their relative configuration remains fixed. During calibration, the laser points are projected onto a reference surface and detected in at least four additional images beyond the standard camera calibration procedure, enabling the estimation of the extrinsic parameters between the laser projector and the camera (Figure 7). The image coordinates of each laser point are extracted by applying Gaussian smoothing followed by parabolic fitting, allowing the laser centers, (u,v), to be localized with subpixel accuracy.

Each plane has a rotation matrix R and a translation vector T, which are estimated for each pose using the checkerboard calibration pattern. The plane normal with respect to the camera is given by $n=R{\left[0,0,1\right]}^{T}$. The intersection of each laser beam with the plane containing the checkerboard calibration pattern is given by

(3) $$n^{⊤}\left(\lambda {\left[\frac{u-c_{x}}{f_{x}},\frac{v-c_{y}}{f_{y}},1\right]}^{⊤}-T\right)=0$$

The reconstructed 3D point is given by

(4) $$X_{3D}=\lambda {\left[\begin{array}{ccc} \frac{u-c_{x}}{f_{x}} & \frac{v-c_{y}}{f_{y}} & 1 \end{array}\right]}^{T}$$

For each laser, several 3D points associated with different planes are obtained. From these points, $X_{k}$, their centroid is computed as

(5) $$\bar{X}=\frac{1}{N}\sum_{k=1}^{N}X_{k}$$

Next, singular value decomposition (SVD) is applied to the matrix formed by the centered points:

(6) $$\tilde{X}=USV^{⊤}$$

A system of equations is formulated for each ray:

(7) p+λd=x

where d is the direction of each laser (obtained from SVD), p is an arbitrary point on the laser (for example, $\bar{X}$), and x is the common intersection point to be found. A linear system is formed:

(8) Ax=b

where *A* is a large matrix composed of identity blocks and direction vectors, and *b* contains the positions p. The laser position with respect to the camera is obtained.

#### 2.2.3. Automatic Detection of Bats

Convolutional neural networks have revolutionized the field of computer vision. Within object detection architectures, the YOLO family (You Only Look Once) stands out. Unlike traditional multi-stage detection methods, YOLO processes the entire image in a single forward pass through the neural network, enabling real-time object detection. Modern versions of YOLO have introduced significant improvements in feature extraction, computational efficiency, and the ability to detect small or partially occluded objects [51,52]. In this work, the YOLO26 architecture is employed, enabling real-time bat detection due to its balance between accuracy, inference speed, and robustness in complex scenarios. In [53], it is reported that YOLO26 introduces important advances in simplicity, speed, and implementation efficiency compared to previous versions. It achieves higher accuracy, particularly for small objects, while also offering a more streamlined implementation and processing speeds up to 43% faster on CPU, making it a suitable architecture for systems with limited computational resources.

#### 2.2.4. Wide-Field Near-Infrared Imaging Platform

Bat monitoring requires a wide field of view because of the high flight speeds of bats and the large dimensions of cave entrances and exits. To address these challenges, we propose a panoramic system composed of one main module and two secondary modules, providing significantly greater spatial coverage than a single camera. The central camera serves as the reference for geometric calibration and image fusion, while the side cameras extend the observable area. After intrinsic calibration and radial distortion correction, the three camera views are registered into a common reference frame, enabling accurate image fusion, continuous target tracking across camera boundaries, and reliable estimation of bat position, velocity, and population density. Let $x_{L}={\left[u_{L},v_{L},1\right]}^{T}$ be a point in the left camera image and $x_{R}={\left[u_{R},v_{R},1\right]}^{T}$ a point in the right camera image. Each camera has its intrinsic matrix:

(9) $$K_{i}=\left[\begin{array}{ccc} f_{x_{i}} & 0 & c_{x_{i}}\\ 0 & f_{y_{i}} & c_{y_{i}}\\ 0 & 0 & 1 \end{array}\right],\;i\in \left\{L,C,R\right\}$$

The pixel is projected into normalized coordinates:

(10) $$x_{i}^{n}=K_{i}^{-1}x_{i}$$

This defines a ray in space:

(11) $$X_{i}=\lambda_{i}x_{i}^{n}$$

where $\lambda_{i}$ is the unknown depth. The rigid transformations are given by

(12) $$T_{CL}=\left[\begin{array}{cc} R_{CL} & t_{CL}\\ 0^{T} & 1 \end{array}\right],\;T_{CR}=\left[\begin{array}{cc} R_{CR} & t_{CR}\\ 0^{T} & 1 \end{array}\right]$$

Then, the points in the central camera coordinate system are

(13) $$\begin{array}{ccc} X_{C}^{\left(L\right)}=R_{CL}X_{L}+t_{CL} & ; & X_{C}^{\left(R\right)}=R_{CR}X_{R}+t_{CR} \end{array}$$

Finally, the points are projected onto the central camera plane:

(14) $$\begin{array}{ccc} x_{C}^{\left(L\right)}=K_{C}X_{C}^{\left(L\right)} & ; & x_{C}^{\left(R\right)}=K_{C}X_{C}^{\left(R\right)} \end{array}$$

Normalizing,

(15) $$x_{C}={\left[\begin{array}{ccc} u_{C} & v_{C} & 1 \end{array}\right]}^{T}=\frac{1}{Z_{C}}{\left[\begin{array}{ccc} X_{C} & Y_{C} & Z_{C} \end{array}\right]}^{T}$$

The complete projection can be written as

(16) $$\begin{array}{ccc} x_{C}^{\left(L\right)}∼K_{C}\left(R_{CL}\lambda_{L}K_{L}^{-1}x_{L}+t_{CL}\right) & ;\; & x_{C}^{\left(R\right)}∼K_{C}\left(R_{CR}\lambda_{R}K_{R}^{-1}x_{R}+t_{CR}\right) \end{array}$$

where ∼ denotes equality up to scale. The calibration of the panoramic system consists of estimating the extrinsic parameters $R_{CL},t_{CL},R_{CR},t_{CR}$ and the intrinsic parameters $K_{L},K_{R},K_{C}$.

## 3. Results

Through different combinations of these modules, the system enables multiple functionalities, including stereo vision, panoramic imaging, and both static and dynamic 3D reconstruction. This design ensures stable, compact, and adaptable operation under diverse requirements for bat monitoring, as it can be deployed in caves where natural illumination is limited or nonexistent.

### 3.1. General System Calibration

**Figure 8:**
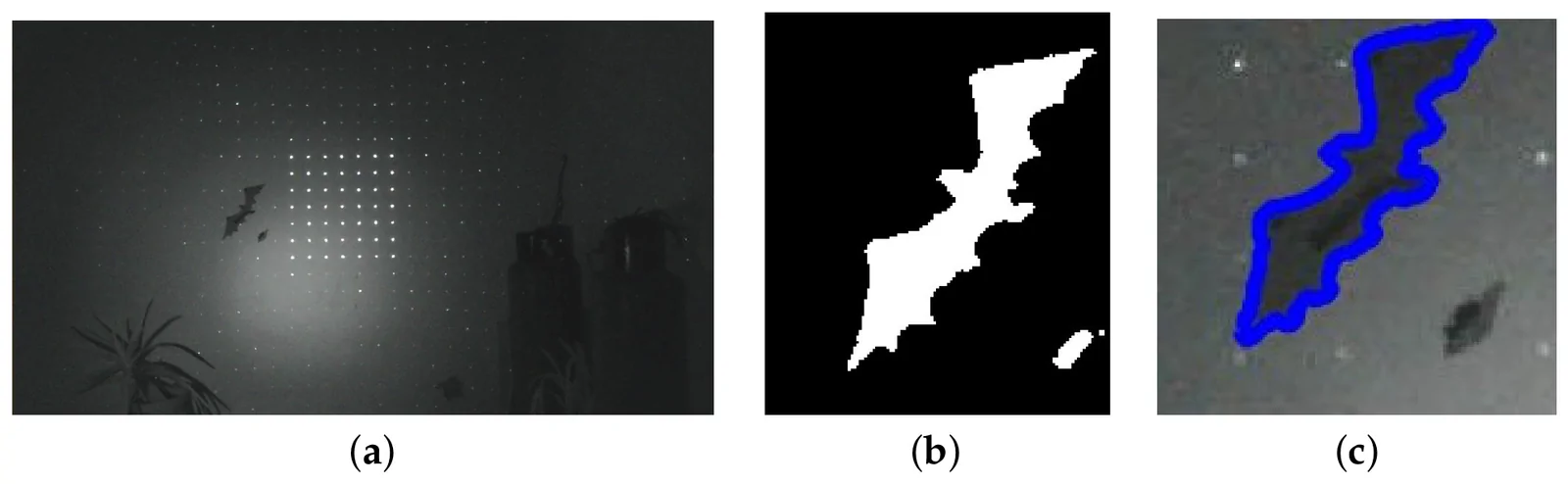
Bat detection process: original image (**a**), preprocessing (**b**), and segmentation result (**c**). The blue color shows the bat’s contour in the figure.

For the proper operation of the modular system, a suitable distance at which the cameras should be positioned was determined. To this end, it is necessary to consider the number of pixels that represent a bat in the image, based on *Desmodus rotundus*. Photographs were taken at various distances ranging from 3 m to 7.5 m. The process is shown in Figure 8.

**Figure 9:**
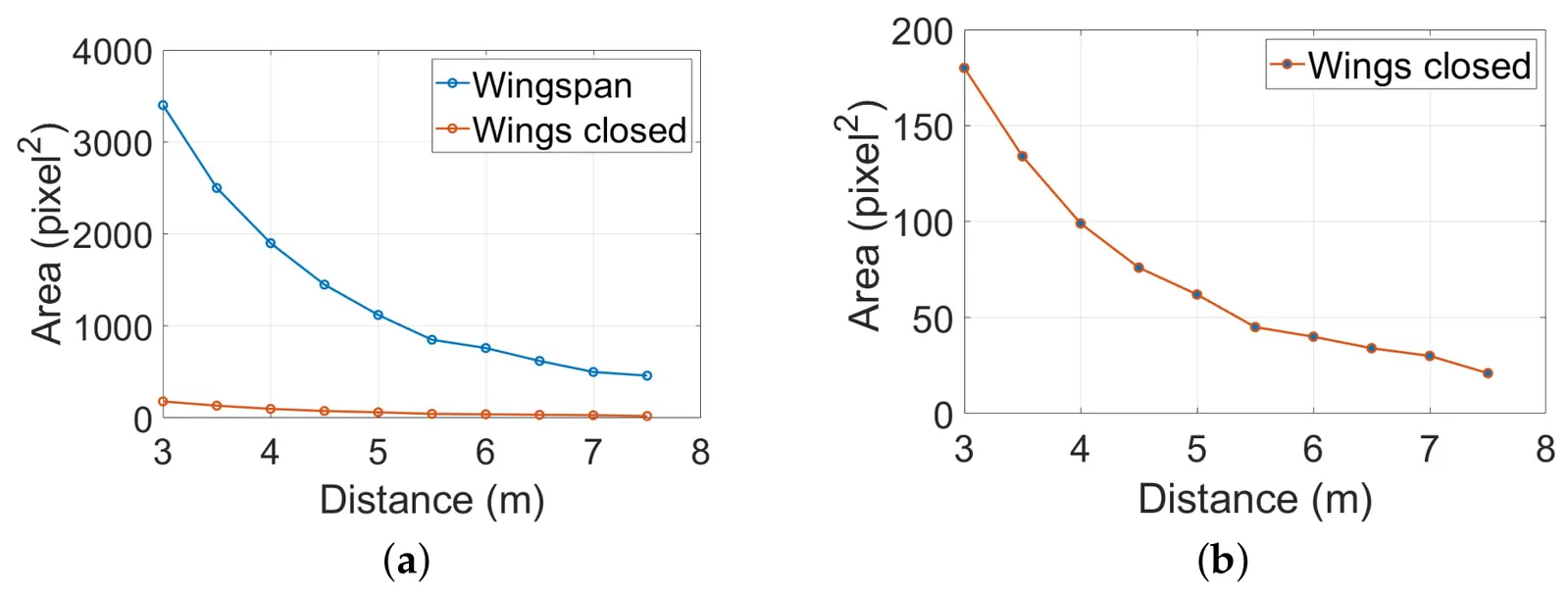
In (**a**), the graph shows the pixel area occupied by a medium-sized bat to determine the appropriate distance at which it can be observed by the camera. In (**b**), the same model is presented with the bat’s wings folded, showing the corresponding pixel area it occupies. With the wings fully extended, the bat occupies approximately 600 pixel^2^, whereas with the wings folded, it occupies approximately 32 pixel^2^.

Figure 9 shows the number of pixels corresponding to the area of bats with wings fully extended and with wings closed, considering that a bat can be detected at distances of up to 8 m from the camera. This figure illustrates that the number of pixels of a *Desmodus rotundus* in flight can be captured by the cameras used without presenting detection issues.

**Figure 10:**
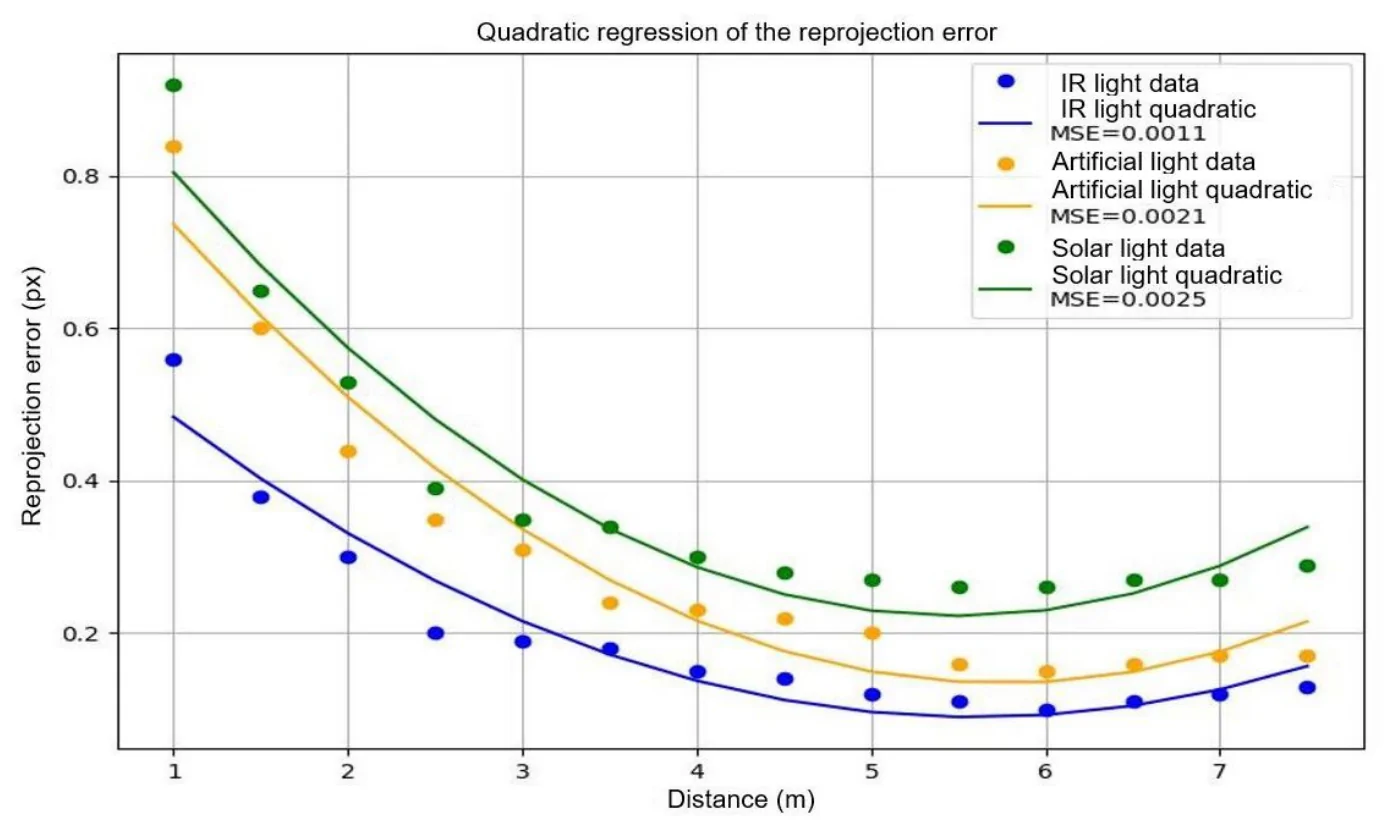
Reprojection error for different calibration configurations under infrared, artificial, and sunlight illumination. The lowest reprojection error was obtained at a camera-to-pattern distance of 6 m under infrared illumination.

To determine the optimal operating configuration, the system was calibrated under different acquisition conditions by varying both the camera-to-scene distance and the illumination source. These experiments were designed to evaluate calibration accuracy and identify the most suitable setup for bat monitoring. The experiments demonstrated that the highest calibration accuracy was obtained under near-infrared illumination at a distance of 6 m. Consequently, this configuration was selected as the nominal operating distance for positioning the sensing modules, as shown in Figure 10.

### 3.2. Dynamic Three-Dimensional Reconstruction of the Environment

This operating mode performs real-time dynamic 3D reconstruction using the Intel RealSense D435i camera integrated into the main module while the operator moves through the environment. Initially, Hector SLAM was evaluated as a complementary 2D mapping approach to improve the accuracy of the 3D reconstruction. However, the experimental results summarized in Table 1 showed that incorporating the additional 2D map did not provide a significant improvement in the final reconstruction accuracy. The cave surfaces contain sufficient natural texture to generate a large number of stable visual features, allowing RTAB-Map to achieve reliable visual odometry, loop closure, and dense 3D reconstruction using only RGB-D data. Consequently, the proposed system relies exclusively on the RGB-D reconstruction pipeline, avoiding unnecessary processing while maintaining high mapping accuracy.

**Figure 11:**
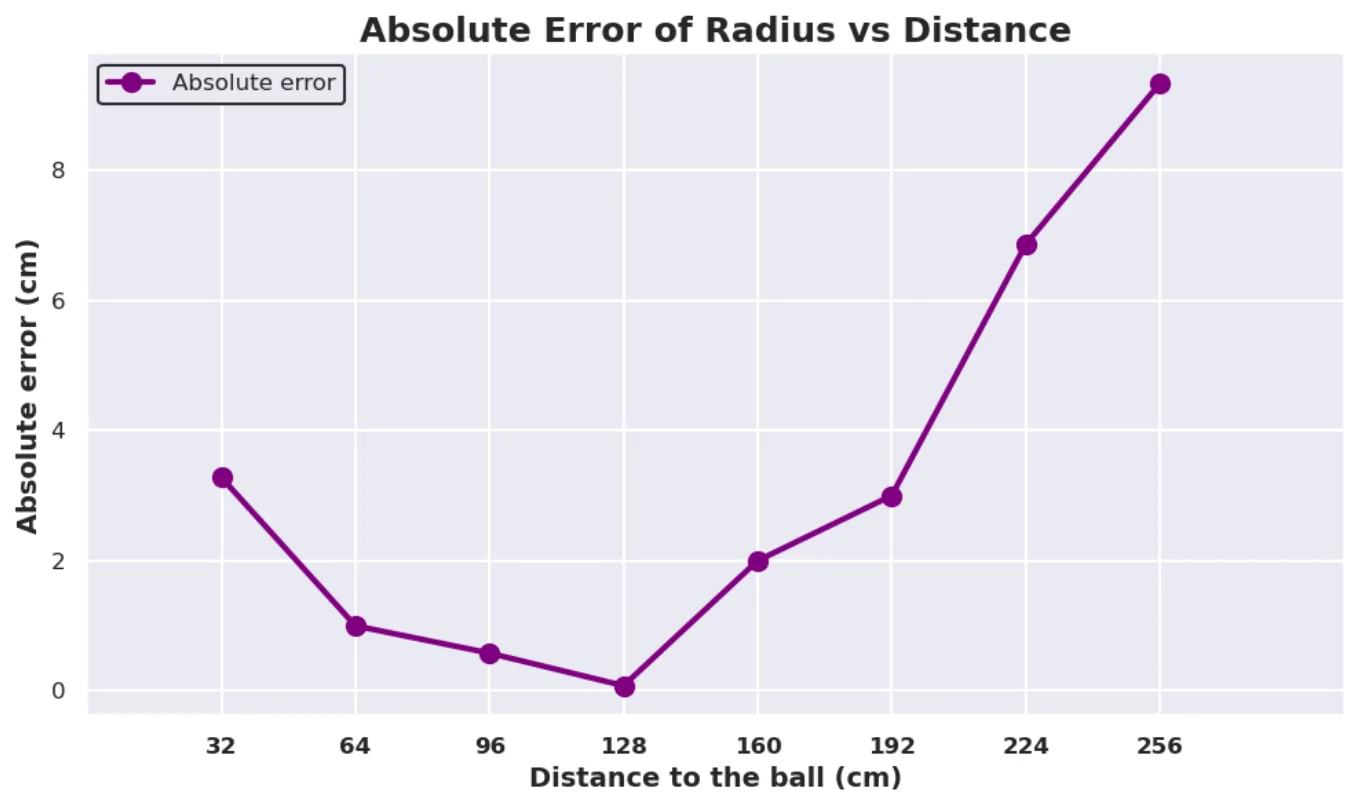
Comparison of the dynamic reconstruction of the sphere. For each distance, datasets were obtained from different viewpoints by moving the module along a circular trajectory centered on the sphere.

**Figure 12:**
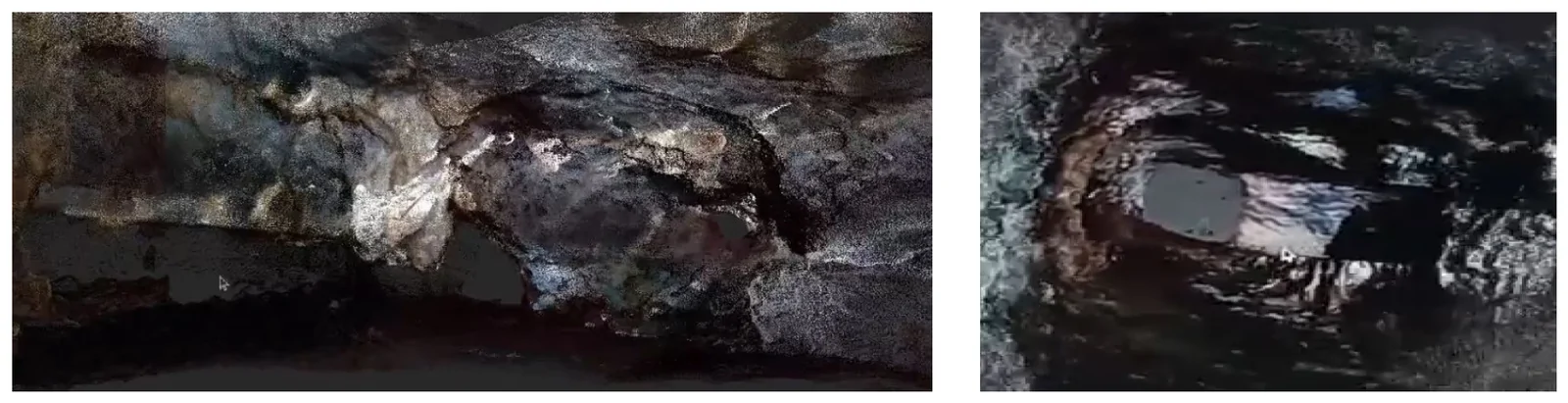
Reconstructed caves. The operator walks through the cave carrying the main module, using the “dynamic three-dimensional reconstruction of the environment” configuration.

Cave reconstructions were carried out following the methodology described in [43]. To evaluate the accuracy of the proposed system, a sphere with a diameter of 66 cm was reconstructed at different acquisition distances, as shown in Figure 11. For each distance, multiple scans acquired from different viewpoints were combined to obtain a complete reconstruction of the sphere. The estimated sphere radius was then used to quantify the reconstruction error. The optimal reconstruction distance was found to be approximately 1.20 m, providing the recommended operating distance between the operator and the cave walls during habitat mapping. Figure 11 presents the corresponding absolute reconstruction error; the sphere was reconstructed using multiple acquisitions captured at a constant distance from different viewpoints. As expected, the reconstruction error increases with distance due to the reduced spatial resolution of the depth sensor. From the obtained results, a cave reconstruction was performed, as shown in Figure 12, resulting in a point cloud that represents the morphology of the caves, contributing to the analysis of bat habitats.

### 3.3. Near-Infrared Stereo Imaging Platform (NISIP)

The NISIP system, composed of a main module and a secondary module, can be calibrated in two ways: using a checkerboard calibration pattern or employing the 49 fixed lasers integrated into the main module. Due to the geometric variability of caves, it is sometimes possible to fix both modules in the area of interest and calibrate the stereo system using a checkerboard pattern. However, we have observed that this approach is not always feasible.

The second strategy for stereo calibration uses the 49 lasers integrated into the main module, which are calibrated beforehand under laboratory conditions. To validate the laser-to-camera calibration, the position of the laser reference system origin, x = (2.6545 −35.5337 ${21.3878)}^{T}$, was estimated with respect to the camera (see Equation (8)) and subsequently compared with a direct physical measurement. This heuristic validation was performed to verify the consistency of the proposed model and the calibration methodology. The estimated distance from the laser origin to the camera, ||x||, was 41.5587 mm, which closely agrees with the measured physical distance. Another test used to validate the correct functioning of the system calibration consisted of reconstructing four planes at different distances.

**Figure 13:**
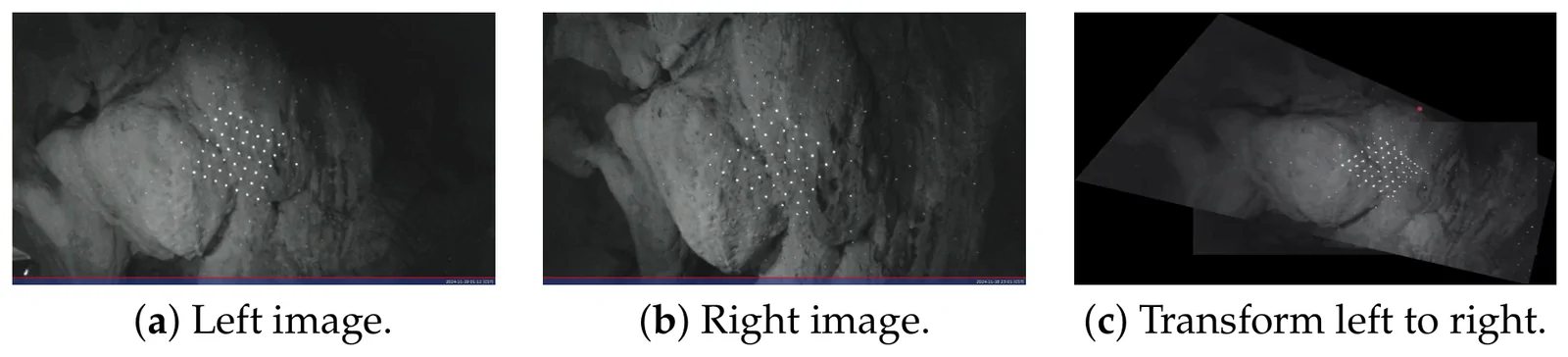
Stereo image generated by the two modules. The transformation validates the estimated intrinsic and extrinsic parameters under in situ operating conditions.

The calibration of the 49 lasers with respect to the main module ensures that the positions captured by the two cameras can be referenced to a common frame using any surface in the real environment, which is necessary for the correct localization and counting of bats. Figure 13a,b show a projection of the lasers onto a cave surface; in this case, it is not possible to use a checkerboard pattern because the cameras have been placed at a wellhead (the upper edge of a shaft or pit). Using this laser projection on the cave wall, the extrinsic parameters between the two cameras are computed. In Figure 13c, this transformation is applied to determine whether the laser points in both images coincide and to assess the consistency of the computed stereo extrinsic parameters.

**Figure 14:**
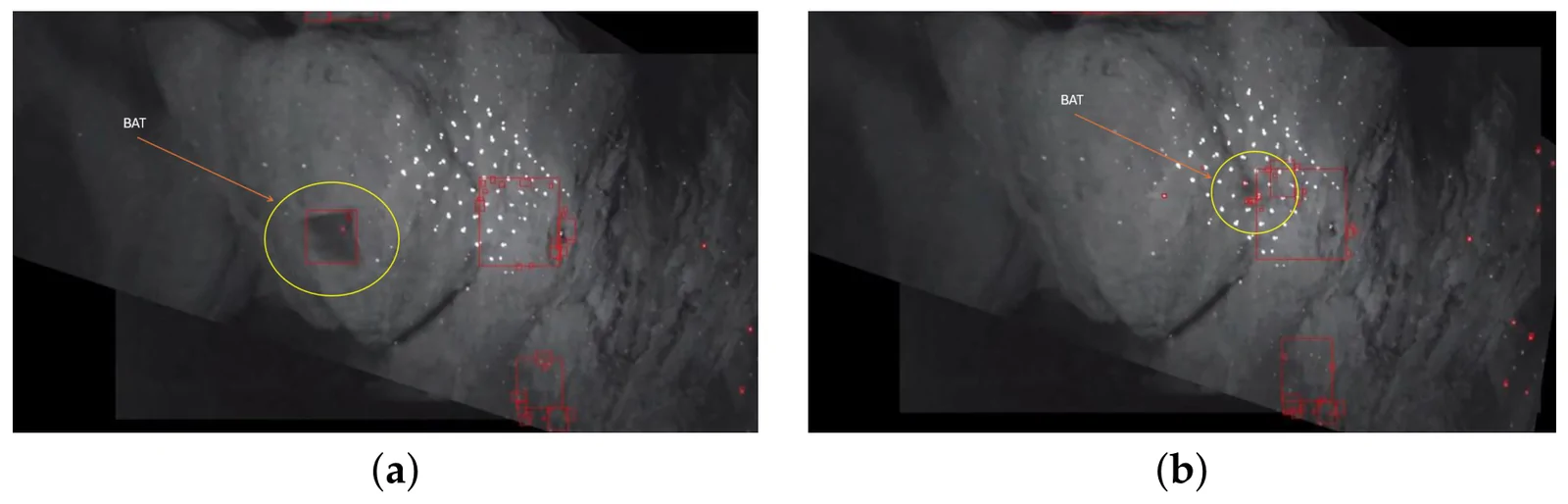
Images acquired by the individual modules at 30 fps. (**a**) Left module. (**b**) Right module. The red box indicates motion detection based on differences between images. The yellow circle indicates a manually detected bat.

For the application of this system, the main module is used together with any of the secondary cameras inside the cave, enabling the estimation of the intrinsic and extrinsic parameters of the second camera through geometric triangulation, thereby generating a global reference system from which three-dimensional environmental data, including information about detected bats, can be obtained. In Figure 14, the image planes are overlaid using triangulation, where each laser point establishes a transformation between the two images. With this, bat velocity between frames can be determined from the change in position over time, as well as flight time.

**Figure 15:**
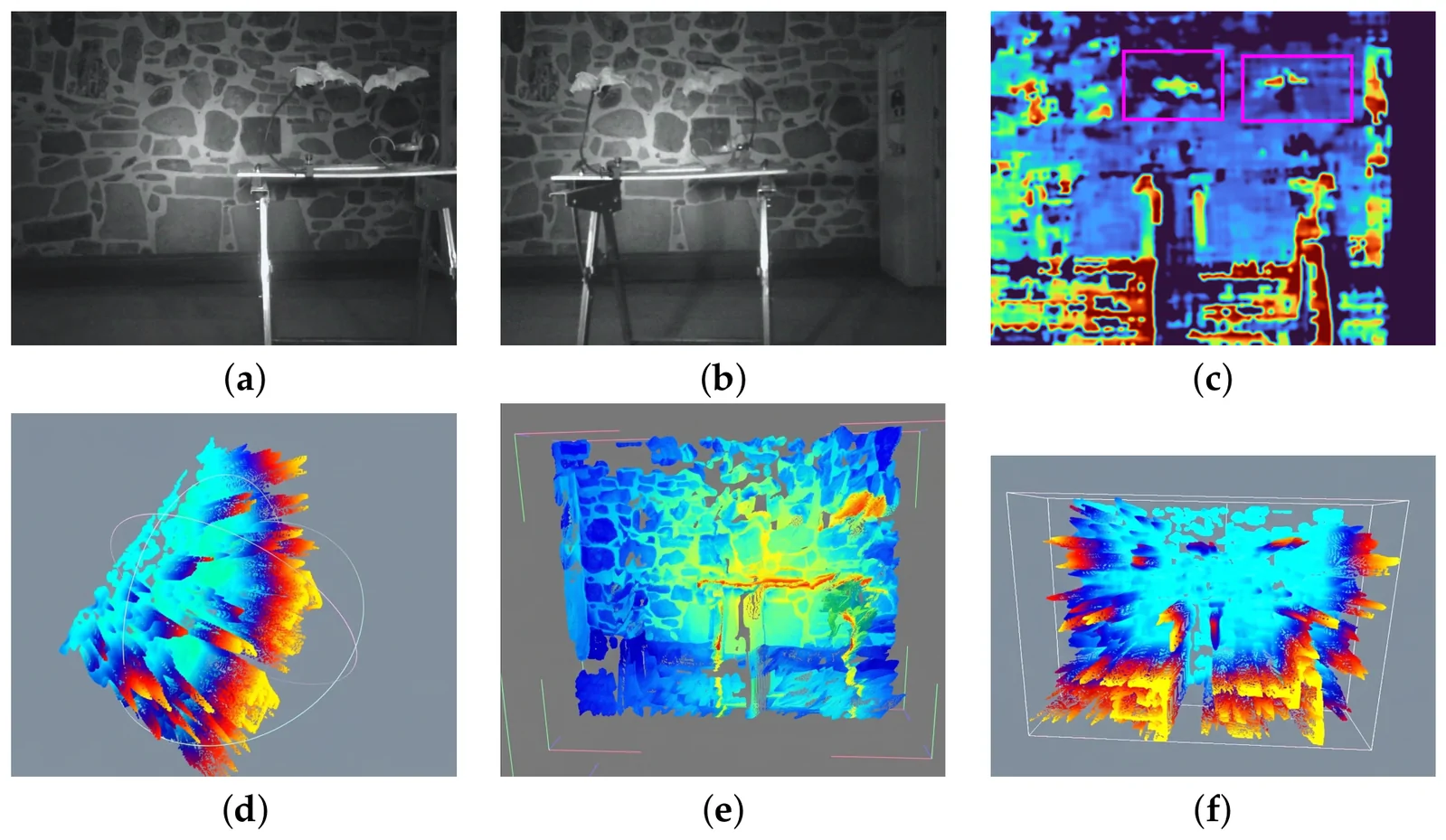
(**a**,**b**) Stereo image pair. (**c**) Disparity image; two dissected bats are highlighted in the two rectangles. (**d**–**f**) Different views of the 3D reconstruction. The colors in Figures (**c**–**f**) represent the *z*-values with respect to the camera. Light blue indicates points with higher *z*-values, whereas yellow indicates points with lower *z*-values.

From the calibrated stereo system, the position of the bat between frames can be determined, showing a displacement of 70 cm in 0.08 s. From this information, the flight speed can be computed, corresponding to 8.75m/s or 31.5km/h. In Figure 15, we can observe the outputs of the Stereo SGBM (**S**emi-**G**lobal **B**lock **M**atching) algorithm projected as a 3D point cloud computed from the stereo system. The first row shows the NIR image pair as well as the resulting depth map obtained by processing this pair with the SGBM algorithm—frames (a) to (c). The second and third rows present projections of this depth map in the open-source software MeshLab 2025.07, which is specialized for point cloud visualization. These figures represent the depth estimates produced by the SGBM algorithm, now projected into three-dimensional space. In the second row, we can see the depth estimates.

### 3.4. Automatic Bat Detection

The bat detection dataset consists of 400 manually annotated near-infrared images containing a total of 427 labeled bats. The images were extracted from videos acquired in five natural caves located in the states of Guanajuato and San Luis Potosí, Mexico, using the proposed imaging platform under representative field conditions. The original dataset contained 365 images from our previously published work, while 35 additional images were incorporated in the present study from the same field expeditions to increase the number of annotated samples.

The dataset was randomly divided into 80% for training and 20% for validation, following the same protocol adopted in our previous work, in which the detector was originally developed and validated. Consequently, images extracted from the same video sequence may be present in both subsets, introducing a potential temporal correlation between the training and validation data. This partitioning strategy was maintained to ensure consistency with the previously validated detector.

Although all images were acquired using the same imaging system, they were collected from multiple natural caves under representative field monitoring conditions, encompassing variations in cave geometry, background texture, bat density, and flight trajectories. In addition, the dataset includes images acquired under different illumination conditions, as some samples were collected using a previous prototype of the monitoring system. These variations increase the representativeness of the dataset for the intended monitoring scenario. Since the primary objective of this work is the integration and validation of the proposed modular multi-sensor monitoring platform, rather than the development of a new bat detection model, the original training protocol was preserved, and no additional data augmentation techniques were applied.

Different variants of the YOLO26 model were evaluated, specifically YOLO26n, YOLO26s, YOLO26m, YOLO26l, and YOLO26x. The comparison was performed using the precision, recall, and the average between both metrics (precision–recall), with the aim of selecting the model that offered the best balance between detection accuracy and object recognition capability. The results obtained are shown in Table 2.

**Figure 16:**
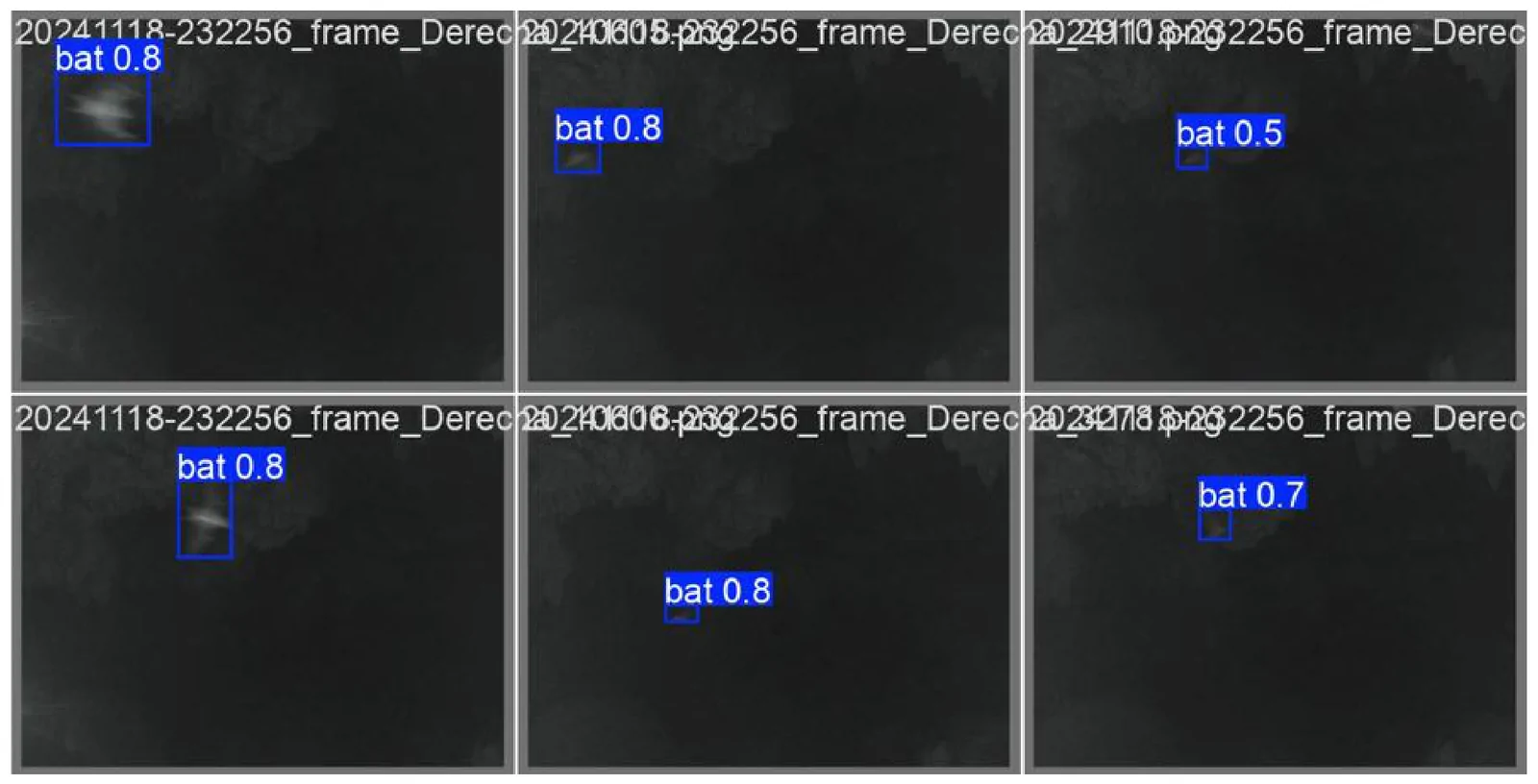
True-positive detection of the bats using YOLO26 l.

**Figure 17:**
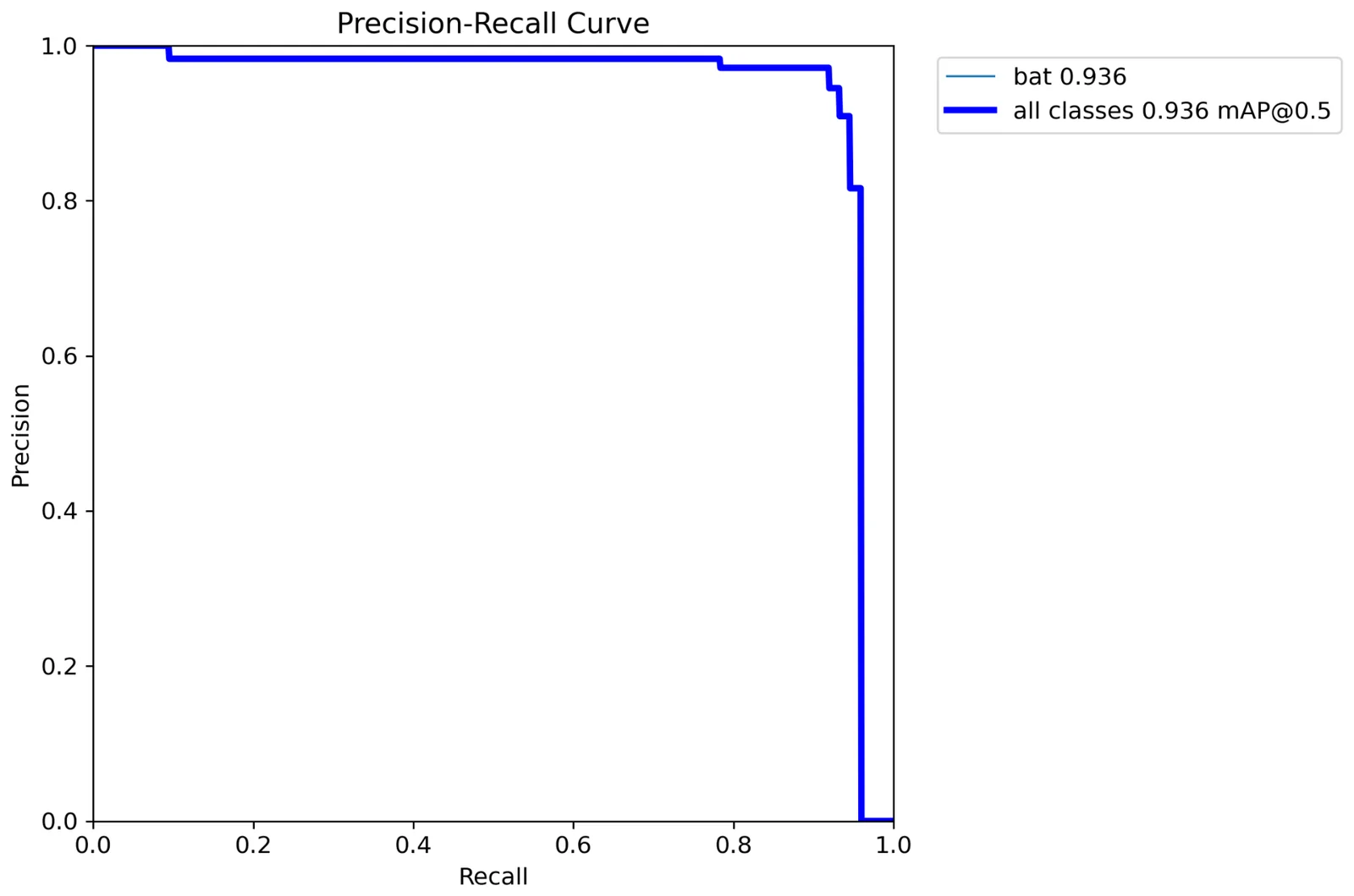
Precision–recall curve. The light blue line is overlapped by the dark blue line.

**Figure 18:**
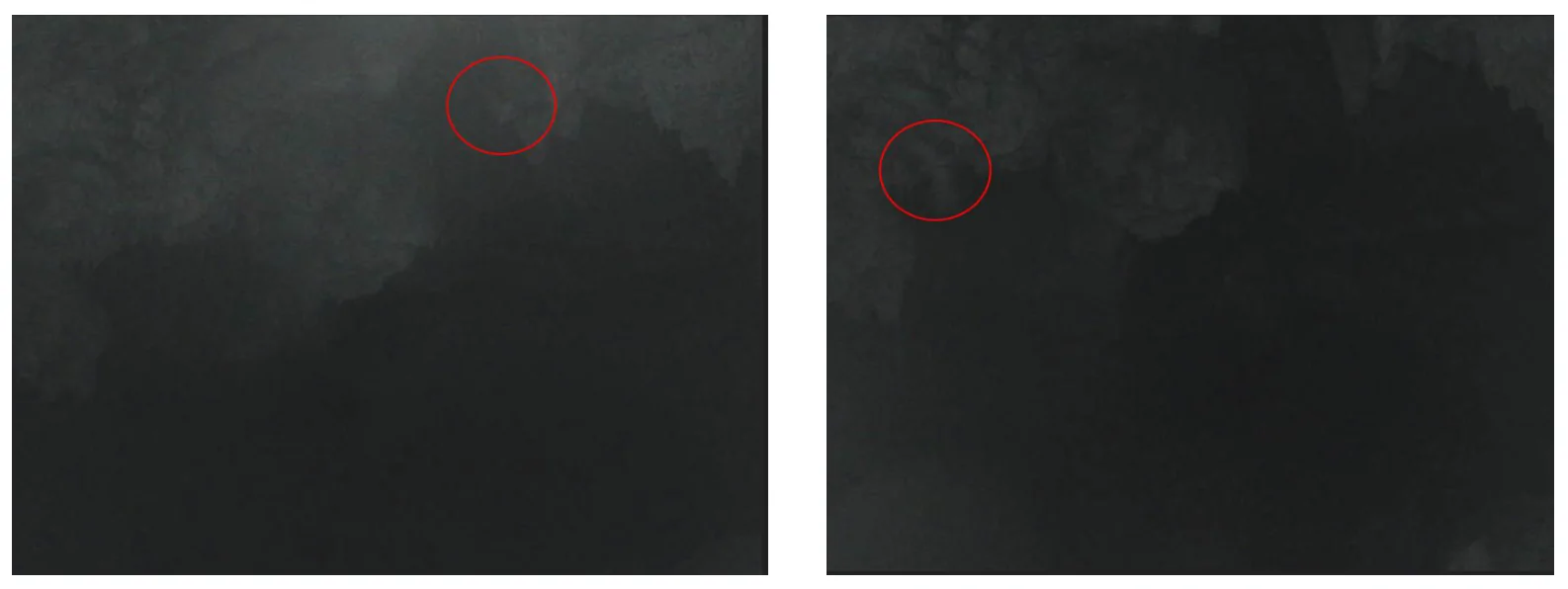
Examples of false negatives, where the system was unable to detect the bats. The bats are indicated with red circles.

Based on the experimental results, the YOLO26l model was selected for bat detection because it provided the best overall balance between precision and recall. Figure 16 presents representative true-positive detection results on the validation set. The selected model achieved a precision of 0.893, a recall of 0.950, and a precision–recall of 0.936 (See Figure 17). These results indicate that the model can accurately detect bats while maintaining a low rate of false positives and false negatives, as illustrated in Figure 18.

### 3.5. Wide-Field Near-Infrared Imaging Platform

**Figure 19:**
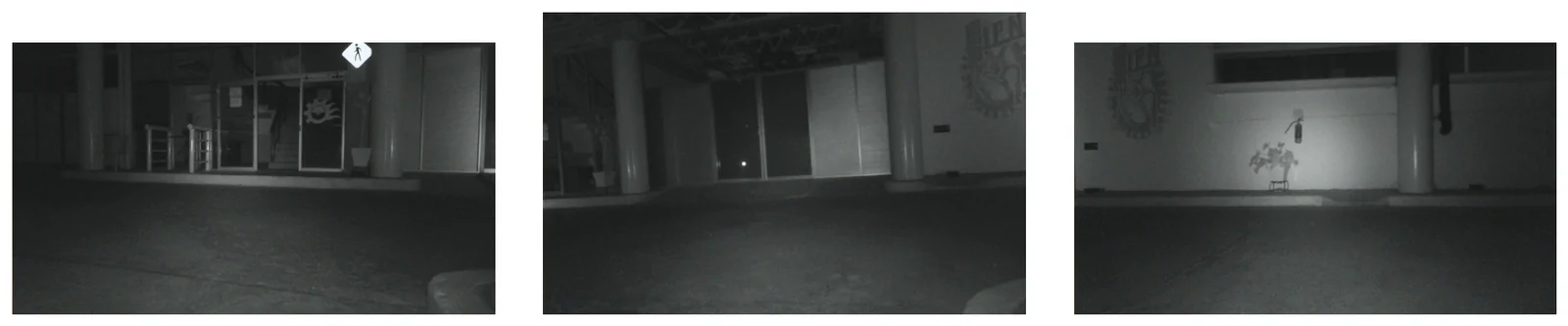
Individual images for panoramic image generation.

Panoramic imaging is essential for bat monitoring in large cave environments, where the field of view of a single camera is insufficient. By combining images from three cameras separated by 7 m, according to the calibration results, the proposed system significantly increases spatial coverage and enables the simultaneous observation of multiple bats (Figure 19). Accurate panoramic image generation requires precise geometric calibration between the cameras, ensuring a common reference frame for all views. This alignment is essential for reliable automatic bat detection and counting, preventing duplicate detections, missed individuals, and counting errors caused by overlapping image regions.

**Figure 20:**
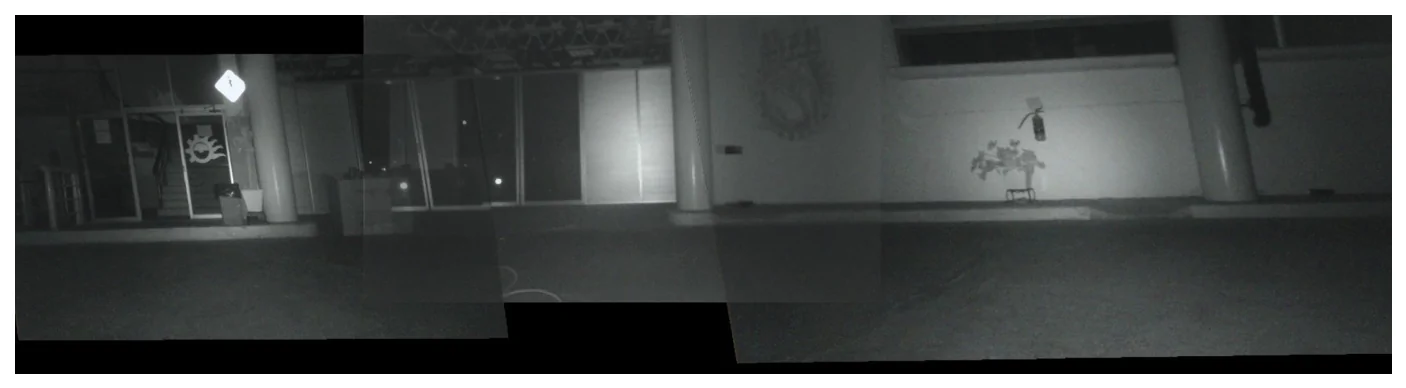
Computed panoramic image.

The result shows a panoramic image generated from the three modules using the previously described configuration, as shown in Figure 20. The panoramic image has dimensions of 720 pixels × 2897 pixels, with a projection error of 0.46 pixels for the left image onto the central image and 0.67 pixels for the right image onto the central image. Considering that our detection objects will have an area greater than 50 pixels, this projection error does not affect the performance of the system. Before generating the panoramic image, radial distortion was removed from the images.

## 4. Discussion

In our previous work, we developed a 3D cave reconstruction system [26] and a YOLO-based bat detection module [43]. In the present work, both functionalities are integrated into the main module, sharing the same hardware and software platform. Furthermore, we evaluate the performance of the 3D reconstruction system under different operating configurations and determine the optimal deployment settings. In addition, the YOLO detector has been updated to a more recent version. Most importantly, we propose a modular configuration of the sensing modules that significantly expands the system’s field of view. This configuration improves bat detection performance by combining panoramic coverage with the updated YOLO model and facilitates the acquisition of a larger and more diverse dataset for future model training. Table 3 summarizes the main differences between our previous work and the proposed system.

### Operating Conditions

The system was designed under a modular and scalable architecture and is therefore not restricted to a specific cave size. Its spatial coverage depends on the number of modules deployed and on the selected operating configuration. In the three-dimensional reconstruction configuration, the horizontal extent of the scene does not constitute a limitation, provided that the operator can move safely within the cave during data acquisition. The main constraint corresponds to the effective reconstruction height, which is determined by the common region of the fields of view of the RGB camera and the depth sensor of the Intel RealSense D435i. According to the manufacturer’s specifications, the depth sensor has an approximate field of view of $87^{\circ }\times 58^{\circ }$, while the RGB camera has a field of view of $69^{\circ }\times 42^{\circ }$ [54]. Considering a working distance of 3 m, the common observable region corresponds to an area of approximately 4.13×2.30 m. Under normal operating conditions, and assuming the system is carried by an operator approximately 1.80 m tall, it is possible to reconstruct surfaces up to 4.10 m in height without changing the system’s position. The stereo configuration was conceived for acquiring three-dimensional information in narrow or hard-to-access areas. During field campaigns, multiple cavities used by bats as entry and exit routes were identified, many of them with small and irregular geometries. In these scenarios, the stereo configuration provides an adequate solution for depth estimation and three-dimensional localization of individuals. The panoramic configuration allows the field of view to be expanded through the incorporation of additional secondary modules. Consequently, the system’s horizontal and vertical coverage can be adapted to the dimensions of the study site, depending solely on the number of available modules and the time allocated for data acquisition. In the automatic detection configuration, the current version of the system uses a single camera. The detection model was trained and validated using the dataset acquired with the system proposed by [43]. However, a quantitative evaluation of its performance during extended operations under natural conditions, including different lighting levels, population densities, and flight trajectories, is part of future work.

Regarding operating conditions, the system can function within the environmental ranges specified by the manufacturers of its components. In particular, the Raspberry Pi 5 supports operating temperatures of up to 70 °C [55]. During the expeditions carried out, the system operated stably in all the caves explored. However, environmental factors such as relative humidity, gas concentration, and the presence of suspended particles could affect its performance during long-duration monitoring campaigns. Consequently, the incorporation of environmental sensors and the evaluation of their influence on system performance constitute a future line of research. Finally, the system does not generate mechanical noise during operation, which minimizes the possibility of altering the natural behavior of the bats. In field tests it was possible to acquire images in situ without observing evident changes in their behavior. However, a quantitative evaluation of the system’s impact on the behavior, flight activity, and response patterns of the colonies will be addressed in future work.

## 5. Conclusions and Future Work

This work presents a scalable multi-sensor vision framework designed for bat monitoring in cave environments using RGB and NIR imaging technologies. The proposed architecture combines modular hardware design, multi-sensor geometric calibration, and panoramic image generation to enhance spatial coverage and improve robustness under low-light conditions. The developed calibration model allows all sensing modules to be represented within a unified three-dimensional reference system through the estimation of intrinsic and extrinsic parameters. This geometric representation ensures consistent spatial relationships among the different cameras and facilitates future three-dimensional reconstruction and localization tasks. Experimental results demonstrate the feasibility of constructing panoramic images from multiple synchronized modules while maintaining low reprojection errors. The obtained projection errors are sufficiently small relative to the target object dimensions, indicating that the proposed system can operate without significantly affecting future automatic bat detection and counting processes. The modularity of the proposed framework enables the integration of additional sensing units according to the dimensions and topology of the monitored environment. This scalability represents an important advantage for applications involving large cave systems or complex subterranean scenarios where a single sensor is insufficient. Future work will focus on integrating automatic bat detection and species identification algorithms based on deep learning, three-dimensional trajectory estimation, and real-time distributed processing for long-term ecological monitoring applications. In future work, we will extend the stereo calibration study by conducting a comprehensive quantitative evaluation of the system’s localization accuracy under different acquisition conditions. The experiments will be performed by placing calibrated targets at known three-dimensional positions over a range of working distances and under both visible and near-infrared illumination. The reconstructed coordinates will be compared with the corresponding ground-truth measurements to quantify localization accuracy using metrics such as the root mean square error (RMSE), mean absolute error (MAE), and maximum localization error. Additional experiments will investigate the influence of camera baseline, module orientation, and nonplanar scene geometry on depth estimation accuracy and calibration stability. The repeatability of the calibration procedure will also be assessed through multiple independent calibration sessions. These experiments will provide a more comprehensive characterization of the system’s performance and further justify the selection of the nominal operating distance adopted in the proposed platform.

## Figures and Tables

**Table 1:** RMSE results (mm) obtained for the generated three-dimensional environment.

System	Fragmented Map (mm)	Complete Map (mm)
Hector SLAM	0.67	0.461
RTAB-Map	40.00	72.09
RTAB-Map Textures	0.78	12.00

**Table 2:** Comparison of performance between YOLO26 variants.

Model	Precision	Recall	Precision–Recall
YOLO26n	0.62	0.97	0.887
YOLO26s	0.90	0.89	0.920
YOLO26m	0.74	0.96	0.928
YOLO26l	0.893	0.95	0.936
YOLO26x	0.76	0.96	0.937

**Table 3:** Comparison of the two multi-sensor bat-monitoring systems.

Aspect	Our Previous Work [26,43]	Scalable Multi-Sensor Framework (Panoramic NIR)
Core focus	Evaluating YOLO detection accuracy on NIR bat imagery	Full modular hardware framework enabling multiple monitoring modes
Architecture	Single portable unit (one integrated device)	Modular: 1 master module + 2 interchangeable slave modules
Operating modes	One: NIR-based detection/counting or 3D cave reconstruction	Four: dynamic 3D reconstruction, stereo 3D bat positioning (NISIP), real-time detection, panoramic wide-field imaging
Cameras	2× Arducam IR cameras (60 fps, 8 cm stereo baseline) + Intel RealSense D435i	Master: Arducam UC844 (1280 × 720 @60 fps) + RealSense D435i; up to 2 additional slave UC844 cameras
Illumination	NIR LED array, 890 nm	9 V IR lamp per module + 49-point laser array (dual-purpose: illumination/calibration)
Onboard compute	Raspberry Pi 4B+ (8 GB RAM)	Master: full computer, 16 GB RAM, 512 GB SSD, Ubuntu 20.04; Slave: Raspberry Pi 5
Weight/size	Not specified in detail (single compact unit)	Total ∼ 6.5 kg—master 3.5 kg (22 × 22 × 14 cm), 2 slaves 1.5 kg each (18 × 18 × 11 cm)
Power/autonomy	∼6 h (5 V + 9.6 V LiPo batteries)	Master ∼ 5 h (dual 19 V Li-ion) + 4 h for IR/laser (9 V); slave ∼ 4.5 h (9 V)
Detection algorithm	YOLOv10, v11, v12 (sizes n/s/m/l/x/b)	YOLO26 (sizes n/s/m/l/x)
Best model & performance	YOLOv12-m: precision 0.956, recall 0.983, mAP@50 = 0.981	YOLO26-l: precision 0.893, recall 0.950, F1 (precision–recall avg) = 0.936
Dataset	Semi-automatically labeled via background subtraction (Gaussian model + online EM), real cave footage, 70/30 train/val split	400 manually curated images, 80/20 train/test split
Statistical validation	Extensive: Friedman test + 480 pairwise Wilcoxon comparisons across all model variants with Holm correction	Not reported
Field deployment	6 caves in Xilitla, San Luis Potosí (species: *Diphylla ecaudata*, *Desmodus rotundus*, *Myotis occultus*)	Lab/controlled validation (sphere reconstruction, wellhead calibration test); no specific cave survey reported
3D/stereo capability	Offline 3D Reconstruction System	Full 3D pipeline: laser-based in situ stereo calibration, SGBM depth maps, bat flight-speed estimation (9.47 m/s measured)
Panoramic imaging	Not included	Yes—3-camera fusion, 720 × 2897 px output, <0.7 px reprojection error, 7 m camera spacing
Environment mapping	Optimal deployment settings not included	RTAB-Map SLAM-based dynamic 3D cave reconstruction (RealSense D435i), validated with 66 cm reference sphere
Novel calibration method	None (standard camera use)	49-point laser array for field-deployable stereo/panoramic calibration without checkerboards
Positioning relative to each other	Earlier/narrower study—validates YOLO detection specifically	Later/broader system—builds on the detection work above (cites it directly) and adds 3D + panoramic capabilities via the modular design

## Data Availability

The original contributions presented in this study are included in the article. Further inquiries can be directed to the corresponding author.

## References

[1] Heim O., Treitler J.T., Tschapka M., Knörnschild M., Jung K. (2015). The Importance of Landscape Elements for Bat Activity and Species Richness in Agricultural Areas. PLoS ONE.

[2] Meraz-Molina K., Luevano-Gurrola S.D., Pinedo-Alvarez A., Villarreal-Guerrero F., Hernández-Quiroz N.S., Ibarra-Bonilla J.S., Fontes-Palma I., Vega-Mares J.H., Prieto-Amparán J.A. (2026). Ecological Corridors for Tadaria brasiliensis in Agricultural Landscapes of Northern Mexico Integrating AHP, InVEST, and Least-Cost Path. Land.

[3] Velásquez-Roa T., Cabrera-Ojeda C., Bernal Rivera A., Calvache Sánchez C., Carvajal-Nieto P., Benavides S., Granobles-Cardona J. (2025). Distribution, ecology, and natural history of the Spectral bat (*Vampyrum spectrum*) in Colombia. Therya.

[4] Ortega-Sánchez R., Bárcenas-Reyes I., Luna-Cozar J., Rojas-Anaya E., Cuador-Gil J.Q., Cantó-Alarcón G.J., Veyna-Salazar N., González-Ruiz S., Milián-Suazo F. (2024). Spatial-temporal Risk Factors in the Occurrence of Rabies in Mexico. Geospat. Health.

[5] Rodríguez-San Pedro A., Allendes J.L., Beltrán C., Chaperon P., Saldarriaga M., Silva A., Grez A. (2020). Quantifying ecological and economic value of pest control services provided by bats in a vineyard landscape of central Chile. Agric. Ecosyst. Environ..

[6] Rosa A., Martorelli F., De A., Kataoka A., Almeida M., Uieda W., Trajano E. (2024). Biology, Shelter Use and Rabies Antibodies in the Hairy-Legged Vampire Bat, *Diphylla ecaudata*, in Cohabitation with *Desmodus rotundus*: A Large Colony in a Man-Made Building in Southeastern Brazil, with Comments on Conservation. Acta Chiropterologica.

[7] Ingala M., Oliveira H., Oliveira M., Frank L., Hussain M., Kazam A., Becker D., Cummings C., Dalannast M., Kingston T. (2025). Bats in Habitats, Bats as Habitats: An integrative ecological framework for understanding synergistic interactions across levels of community organization. EcoEvoRxiv.

[8] Rojas-Sereno Z., Streicker D., Yung V., Broos A., Malik H., Bergner L., Lineros M., Benavides J. (2026). Rabies, host population structure, and cross-species transmission to the migratory bat Tadarida brasiliensis in Chile. PLoS Negl. Trop. Dis..

[9] Gudiño-Escandón R.S., Méndez-Ojeda M.L., García-Sarabia S.P., Luna-Azuara C.G., Vega Murillo V.E. (2025). Pérdida económica por rabia paralítica en bovinos y especies ganaderas de México en 2022. Rev. Mex. Cienc. Pecu..

[10] Kunz T.H., Betke M., Hristov N.I., Vonhof M.J., Kunz T.H., Parsons S. (2009). Methods for Assessing Colony Size, Population Size, and Relative Abundance of Bats. Ecological and Behavioral Methods for the Study of Bats.

[11] Elphick C.S. (2008). How You Count Counts: The Importance of Methods Research in Applied Ecology. J. Appl. Ecol..

[12] Moosman P.R., Marsh D.M., Pody E.K., Dannon M.P., Reynolds R.J. (2020). Efficacy of Visual Surveys for Monitoring Populations of Talus-Roosting Bats. J. Fish Wildl. Manag..

[13] Yan W.Q. (2026). Image Processing for Robotics. Robotic Vision: From Deep Learning to Autonomous Systems.

[14] Rong Z., Cong S., Tang L., Tian S., Goh S.H., Sheng L., Nie Y. (2026). Infrared-based real-time leakage detection in earth–rock dams using an enhanced YOLO algorithm integrated with a biomimetic quadruped robot. Appl. Soft Comput..

[15] Arumugam V., Alagumalai V. (2026). Enhancing UAV Autonomy and Path Optimization Through SBAS and Visual-Inertial Odometry. Ing. Investig..

[16] Che C., Zheng H., Huang Z., Jiang W., Liu B. (2024). Intelligent Robotic Control System Based on Computer Vision Technology. arXiv.

[17] Geng Y., Zhang Y., Tian X., Zhou L. (2024). A novel 3D vision-based robotic welding path extraction method for complex intersection curves. Robot. Comput.-Integr. Manuf..

[18] Nivethika S.D., Joshi S., Verma K., Aishwarya V., Srinivasan V.V., Pandian M.S., Paulraj P. (2026). Attention-guided spatio-temporal feature fusion for robust video surveillance anomaly detection. Sci. Rep..

[19] Suárez-Pesántez G.F., Vizñay-Durán J.K. (2021). Desarrollo de un sistema de alarma domiciliaria con reconocimiento facial y alerta temprana. Caso de estudio: Vivienda del Barrio Corazón de María, Cantón Cuenca, Provincia del Azuay. Polo Conoc..

[20] Sesha S.E. (2025). Computer Vision Applications for Enhanced Warehouse Safety: A Comprehensive Analysis of Load Securing and Weight Management Systems. J. Comput. Sci. Technol. Stud..

[21] Liu X., Tang Z., Wang W., Chen Z., Du Z., Zhai R., Yuan Y., Dong X., Chen Q., Cheng Y. Smart Warehouse Monitoring System for Collaborative Character Recognition Platform Research. Proceedings of the 2024 8th Asian Conference on Artificial Intelligence Technology (ACAIT).

[22] Chen F., Jingliang S., Yongliang L., Jia Y., Lin L., Yubin S., Lei Z., Shaotong P. (2026). Based on Improved YOLOv11 Transmission Line Bird Hazard Detection. J. Sens..

[23] Antoniazzi R., Velasquez-C. K., Menzel E.R., Stage D., Hughes K., Andrade-Ponce G. (2026). Climbing the urban canopy: Camera trap insights into mammal activity and habitat use. Wildl. Soc. Bull..

[24] Bohnett E., Lamichanne B.R., Chaudhary S., Pokhrel K., Coulter L., Dormann G., Flores A., Lewison R.L., Qiu F., Stow D. (2026). Understanding the efficacy and efficiency of thermal infrared UAV for wildlife monitoring. Environ. Monit. Assess..

[25] Yoshida S., Matsuda Y., Tsubouchi K., Suwa H., Yasumoto K. Wildlife Detection using Motion History Information Captured by Camera Trap in the Dark. Proceedings of the 27th International Conference on Distributed Computing and Networking, ICDCN Companion ’26.

[26] Cruz Rangel I., Arroyo-Romero J.A., Bárcenas-Reyes I., González-Barbosa J.J., Hurtado-Ramos J.B., Ornelas-Rodríguez F.J., Ramírez-Pedraza A. (2025). Explorando las Profundidades: Reconstrucción de Cuevas y Detección de Murciélagos mediante Imágenes Infrarrojas. Mem. Investig. Ing..

[27] Jayathunga D.P., Ramashini M., Zaini J., Müller R., De Silva L.C. (2026). Improved YOLOv5 for Efficient Elimination of Unwanted Video Frames From High-Speed Video Arrays Capturing Bat’s Kinematics. Appl. AI Lett..

[28] Weaver S.P., Ritter J.D., Commiskey A.M., Garcia J.D., Morton B.P. (2025). Testing a bat fatality detection system at wind turbines. PLoS ONE.

[29] Teshima Y., Hayashi Y., Aoki Y., Fujioka E., Hiryu S., Kawagucci S. (2026). evsBat: An automated toolkit for tracking and quantifying rapid movement of nocturnal animals using event cameras. Methods Ecol. Evol..

[30] Ahlberg S.E., Kuczynska V., Kloepper L.N. (2025). A Perspective on Thermal Imagery for Bat Emergence Counts: Best Practices, Challenges, and Recommendations for the Future. J. N. Am. Bat Res..

[31] Fujioka E., Fukushiro M., Ushio K., Kohyama K., Habe H., Hiryu S. (2021). Three-Dimensional Trajectory Construction and Observation of Group Behavior of Wild Bats During Cave Emergence. J. Robot. Mechatron..

[32] McCarthy E.D., Martin J.M., Boer M.M., Welbergen J.A. (2021). Drone-based thermal remote sensing provides an effective new tool for monitoring the abundance of roosting fruit bats. Remote Sens. Ecol. Conserv..

[33] Happ C., Sutor A., Hochradel K. (2024). UAV-Based 3D-Calibration of Thermal Cameras for Bat Flight Monitoring in Large Outdoor Environments. Remote Sens..

[34] Krivek G., Gillert A., Harder M., Fritze M., Frankowski K., Timm L., Meyer-Olbersleben L., von Lukas U.F., Kerth G., van Schaik J. (2023). BatNet: A deep learning-based tool for automated bat species identification from camera trap images. Remote Sens. Ecol. Conserv..

[35] Bethsabe O.H., Sanchez Garcia A., Delfín-Alfonso C.A., Ocharán-Hernández J., Morteo E., H.V. F.R. (2018). Desarrollo de una aplicación para el conteo automático de murciélagos en cuevas basado en visión por computadora. Res. Comput. Sci..

[36] Bentley I., Gebran M., Vorderer S., Ralston J., Kloepper L. Utilizing Neural Networks to Resolve Individual Bats and Improve Automated Counts. Proceedings of the 2023 IEEE World AI IoT Congress (AIIoT).

[37] Koger B., Hurme E., Costelloe B.R., O’Mara M.T., Wikelski M., Kays R., Dechmann D.K.N. (2023). An automated approach for counting groups of flying animals applied to one of the world’s largest bat colonies. Ecosphere.

[38] Darras K., Yusti E., Huang J.C.C., Zemp D., Kartono A., Wanger T. (2021). Bat point counts: A novel sampling method shines light on flying bat communities. Ecol. Evol..

[39] Darras K.F.A., Balle M., Xu W., Yan Y., Zakka V.G., Toledo-Hernández M., Sheng D., Lin W., Zhang B., Lan Z. (2024). Eyes on nature: Embedded vision cameras for terrestrial biodiversity monitoring. Methods Ecol. Evol..

[40] Lee B., Rich A., Diehl R.H., Larsen A. (2024). BATS: Bat-aggregated time series—A python-based toolkit for landscape-level monitoring of free-tailed bats via weather radar. Methods Ecol. Evol..

[41] Crawford R.D., O’Keefe J.M. (2024). Improving the science and practice of using artificial roosts for bats. Conserv. Biol..

[42] Zubair A., Mukhtar R., Ahmed H., Ali M. (2024). Emergencies of zoonotic diseases, drivers, and the role of artificial intelligence in tracking the epidemic and pandemics. Decod. Infect. Transm..

[43] González-Barbosa J.J., Rangel I.C., Ramírez-Pedraza A., Ramírez-Pedraza R., Bárcenas-Reyes I., González-Barbosa E.A., Razo-Razo M. (2025). Deep Learning for Wildlife Monitoring: Near-Infrared Bat Detection Using YOLO Frameworks. Signals.

[44] Rowse E.G., Lewanzik D., Stone E.L., Harris S., Jones G., Voigt C.C., Kingston T. (2016). Dark Matters: The Effects of Artificial Lighting on Bats. Bats in the Anthropocene: Conservation of Bats in a Changing World.

[45] Gnocchi A.P., Srbek-Araujo A.C. (2017). Common Vampire Bat (*Desmodus rotundus*) feeding on Lowland Tapir (*Tapirus terrestris*) in an Atlantic Forest remnant in southeastern Brazil. Biota Neotrop..

[46] González M.E., Sierra O.H., Higuera O.I. (2025). Active Disturbance Rejection Control of a DC Brushed Motor Using Simulink and Raspberry Pi. Ing. Investig..

[47] Díaz-Delgado D., Inga Alva A.E. (2025). Development of a Solid Waste Collector Robot for Cleaning in Public Areas. Ing. Investig..

[48] Tsykunov E., Ilin V., Perminov S., Fedoseev A., Zainulina E. (2020). Coupling of localization and depth data for mapping using Intel RealSense T265 and D435i cameras. arXiv.

[49] Labbé M., Michaud F. (2013). Appearance-based loop closure detection for online large-scale and long-term operation. IEEE Trans. Robot..

[50] Labbé M., Michaud F. (2019). RTAB-Map as an Open-Source Lidar and Visual Simultaneous Localization and Mapping Library for Large-Scale and Long-Term Online Operation. J. Field Robot..

[51] Li Y., Li Q., Pan J., Zhou Y., Zhu H., Wei H., Liu C. (2024). SOD-YOLO: Small-Object-Detection Algorithm Based on Improved YOLOv8 for UAV Images. Remote Sens..

[52] Li P., Wen M., Zeng Z., Tian Y. (2025). Cherry Tomato Bunch and Picking Point Detection for Robotic Harvesting Using an RGB-D Sensor and a StarBL-YOLO Network. Horticulturae.

[53] Ultralytics (2026). YOLO26 Documentation. https://docs.ultralytics.com/models/yolo26/.

[54] Intel Corporation (2024). Intel^®^ RealSense™ Depth Camera D435i Specifications. https://www.intel.com/content/www/us/en/products/sku/190004/intel-realsense-depth-camera-d435i/specifications.html.

[55] Raspberry Pi Ltd. (2025). Raspberry Pi 5 Product Brief.

